# Partial Replacement of Fish Meal with Tuna By-Product Meal, Black Soldier Fly Larvae Meal, Fermented Soybean Meal, and Their Two Combinations in Olive Flounder (*Paralichthys olivaceus*) Diets: Effects on Growth, Feed Utilization, Biochemical Composition, and Economic Analysis

**DOI:** 10.3390/ani16213538

**Published:** 2026-11-09

**Authors:** Jabed Hasan, Sung Hwoan Cho, Taeho Kim

**Affiliations:** 1 Department of Convergence Interdisciplinary Education of Maritime and Ocean Logistics, Korea Maritime and Ocean University, Busan 49112, Republic of Korea; hasanj@g.kmou.ac.kr; 2 Division of Convergence on Marine Science, Korea Maritime and Ocean University, Busan 49112, Republic of Korea; 3 Department of Marine Production Management, Chonnam National University, Yeosu 59626, Republic of Korea; kimth@chonnam.ac.kr

**Keywords:** fish meal substitution, alternative protein source, fishery by-product meal, combination of animal and plant protein sources, PCA, sustainable aquaculture

## Abstract

Fish meal (FM) remains a primary protein source in aquafeeds; however, its high cost and limited availability necessitate the exploration of alternative protein sources. This study, thus, investigates the effects of replacing 10% FM protein, equivalent to 22.2% of FM, with tuna by-product meal (TBM), black soldier fly (*Hermetia illucens*) larvae meal (BSF), fermented soybean meal (FSB), and their two combinations in diets on the growth, feed utilization, and biochemical composition of olive flounder (*P. olivaceus*), as well as on economic efficiency. A total of 450 juveniles averaging 10.3 g were randomly distributed into 18 300 L flow-through tanks (25 juveniles per tank). In the control (Con) diet (45% FM-basal), 10% FM protein was replaced with TBM, BSF, FSB, or a combination of TBM and BSF or TBM and FSB at a 1:1 ratio, referred to as the TBM, BSF, FSB, CTB, and CTF diets, respectively. Each experimental diet was randomly allocated to three replicate groups of fish. The fish were hand-fed to apparent satiation twice daily for 12 weeks. The weight gain, specific growth rate, feed consumption, and feed efficiency of fish fed the TBM diet were comparable to those fed the Con diet, but significantly higher than those fed the BSF, FSB, CTB, and CTF diets. The protein efficiency ratio of fish fed the TBM diet was significantly higher than that of fish fed the FSB diet. The protein retention of fish fed the TBM diet was significantly higher than that of fish fed the FSB and CTF diets. The condition factor of fish fed the Con and TBM diets was significantly higher than that of fish fed all other diets. In contrast, dietary treatment had no significant influence on the viscerosomatic index, hepatosomatic index, blood chemistry, proximate composition, and amino acid profiles of the fish. The economic conversion ratio was the lowest in the TBM diet, while the Con and TBM diets led to a significantly higher economic profit index than all other diets. Among the five tested formulations, thus, TBM was the best-performing substitute for FM when 10% FM protein was replaced in a 45% FM-basal diet of olive flounder.

## Simple Summary

The high cost and limited availability of fish meal (FM) have driven the search for alternative protein sources in aquafeeds. FM in the diet of olive flounder (*P. olivaceus*) was therefore partially (10% FM protein, being equivalent to 22.2% of FM) replaced with tuna by-product meal (TBM), black soldier fly (*Hermetia illucens*) larvae meal (BSF), fermented soybean meal (FSB), and their two combinations (TBM and BSF or TBM and FSB = 1:1) to investigate their effects on growth, feed utilization, and economic efficiency. Fish fed the TBM diet had comparable weight gain, specific growth rate, feed consumption, and feed efficiency to those fed the Con diet, while fish receiving these diets outperformed fish receiving the BSF, FSB, and two combined diets. The dietary treatments exhibited no significant effects on the blood chemistry, proximate composition, and amino and fatty acid profiles of the fish, except for C12:0, C18:0, and C22:1n-9. The Con and TBM diets produced a significantly higher economic profit index than all other diets. Conclusively, TBM was the best-performing alternative protein source for FM among the five tested formulations at 10% FM protein replacement in a 45% FM-based diet of olive flounder.

## 1. Introduction

Olive flounder (*P. olivaceus*), a highly esteemed flatfish across Eastern Asia, particularly in Korea, inhabits the coastal waters of the northwest Pacific, spanning from the South China Sea to the Kuril Islands [1]. Initially, it predominantly consumes mysid crustaceans during its larval and juvenile phases, thereafter shifting to a piscivorous diet, adeptly preying on small wild fish as it matures in the wild [2]. In 2024, the aquaculture of olive flounder achieved a yield of approximately 40,125 metric tons (MT), representing 49% of total aquaculture production and an estimated value of 684 billion KRW in Korea [3]. Aquaculture of this species is increasingly adopted owing to its desirable flavour, robust growth, adaptability to variable culture environments, resilience against pathogens, and high market demand [4,5]. Dietary protein requirements for olive flounder vary from 40% to 60%, contingent upon life stages [2], with fish meal (FM) constituting approximately 50–70% of the diet [6]. Feed expenditure can represent up to 70% of the total cost in aquaculture, with high FM inclusion being a significant cost driver [7]. FM is highly digestible and palatable, providing indispensable amino acids (IAAs), long-chain n-3 fatty acids, essential vitamins, and bioavailable minerals, thus serving as a cornerstone of aquafeeds [8]. Nevertheless, the escalating demand from global aquaculture expansion, coupled with the limited and fluctuating supply of FM, has substantially increased its cost. This situation underscores the urgent need to look for alternative protein sources that are economically viable, environmentally sustainable, and consistently available year-round [8,9].

Seafood by-product meal is emerging as a sustainable and economically viable alternative to FM in aquafeeds [10]. Tuna by-product meal (TBM), consisting of tuna heads, bones, viscera, blood, skin, fins, and residual flesh from the canning industry, is widely adopted in aquafeeds, effectively repurposing the substantial waste (up to 65–70%) stream generated by tuna processing [11]. TBM exhibits relatively high levels of crude protein and lipid content, functioning as an effective partial or complete substitute for FM in various carnivorous fish species, such as rockfish (*Sebastes schlegeli*) [12,13], red sea bream (*Pagrus major*) [14], and spotted rose snapper (*Lutjanus guttatus*) [15].

In recent years, insect meal has gained prominence and recognition as a viable alternative to FM in aquafeeds, due to its capacity to convert organic food waste into valuable protein ingredients for aquaculture [16,17]. Black soldier fly (*Hermetia illucens*) larvae meal (BSF) features excellent nutritional value, high digestibility, and a reduced ecological footprint, making it a viable and high-performing substitute for conventional FM [18] in various carnivorous fish species including Atlantic salmon (*Salmo salar*) [19], barramundi (*Lates calcarifer*) [20], Japanese seabass (*Lateolabrax japonicus*) [21], turbot (*Scophthalmus maximus* L.) [22] and tongue sole (*Cynoglossus semilaevis*) [23].

Fermentation is an environment-friendly and cost-effective processing method, benefiting aquaculture sustainability [24,25]. Fermented soybean meal (FSB) is a processed form of soybean meal, where microbes break down harmful compounds and modify complex macromolecules, which ultimately enhances nutrient quality into a more digestible functional ingredient [26]. Fermentation generally improves soybean meal by reducing anti-nutritional factors (ANFs), such as phytic acid, trypsin inhibitors, glycinin, and β-conglycinin, while simultaneously improving digestibility, protein quality, and palatability [27,28]. FSB has been recognized as an alternative to FM in several carnivorous fish species, such as black seabream (*Acanthopagrus schlegelii*) [29], coho salmon (*Oncorhynchus kisutch*) [30], largemouth bass (*Micropterus salmoides*) [31,32], and pompano (*Trachinotus ovatus*) [33].

Substituting high levels of FM with alternative protein sources often diminishes feed palatability, reduces voluntary feed intake, and eventually deteriorates the growth performance of fish [34,35]. Specifically, at elevated levels of FM substitution with an alternative animal protein or plant protein, fish diets often fail to meet the IAA requirements due to the limited IAA profiles of this single source [36,37]. Arginine, lysine [38] and methionine [39] among IAAs are likely to be lacking or deficient when excessive FM is replaced with an alternative protein source in aquafeeds. However, a partial replacement of FM with an alternative protein source in aquafeeds can help to maintain the nutritional adequacy of a diet while reducing reliance on FM as well as facilitating an assessment of the biological responses of fish to an alternative source [40]. Therefore, selections of appropriate ingredient combinations and amino acid balances are important considerations during diet formulation with alternative protein sources for FM.

The substitutability of FM protein up to 18% was achieved by fermented TBM [41] in a 7-week feeding trial and FSB [42] in an 8-week feeding trial in the 60% FM-basal diets of olive flounder (mean initial weight of 12.6 g and 11.9 g, respectively). In addition, FM protein replacement up to 10% with full-fat BSF was feasible in practical diets formulating a 45% FM-basal diet of olive flounder (mean initial weight of 34.3 g) in a 15-week feeding trial [43]. Although these findings indicated the substitutability of FM protein at a moderate level, the differences in dietary formulation and basal FM levels adopted would disturb direct comparison in the efficacy of these alternative protein sources as well as their applicability in the practical diets of olive flounder. In addition, the application of combined alternative protein sources as an FM replacer rather than a single alternative protein source in aquafeeds can improve their substitutability because the combined protein source compensates for deficient nutrients in a single protein source [44]. Furthermore, combining alternative protein sources increases the palatability of the diets, which leads to enhanced growth performance and feed consumption (FC) by fish [45,46].

However, the substitutability of FM protein with a single alternative protein source including TBM [41], BSF [43], and FSB [42] at a moderate level have been investigated in diets of olive flounder by evaluating growth, feed utilization, and physiological responses. The combined use of these single alternative protein source and their associated economic analysis in the same experimental system remain limited. This study, therefore, aims to assess the effects of these three alternative protein sources and their two combinations at a partial replacement level in practical diets on the growth, feed utilization, and biochemical composition of olive flounder, and economic analysis.

## 2. Materials and Methods

### 2.1. Experimental Design

Juvenile olive flounder of similar size, bought from a hatchery (Tongyeong-si, Gyeongsangnam-do, Korea), were transferred to the Institute of Ocean Science Education (Marine Research Unit, building 4), Gangneung-Wonju National University (Gangneung-si, Gangwon-do, Korea), in accordance with established protocols. Initially, the fish underwent a 10-day acclimation period in a 5-ton circular flow-through water tank with a volume of 3.5 tons. The fish were fed a commercial extruded pellet (crude protein: 56%; crude lipid: 8%) (National Federation of Fisheries Cooperatives Feed, Uiryeong-gun, Gyeongsangnam-do, Korea) twice daily at a ratio of 2–3% of total biomass throughout the acclimation period.

Subsequently, 450 robust juveniles (initial body weight of 10.3 ± 0.00 g, mean ± SE) were randomly allocated to 18 300 L circular flow-through tanks (water volume of 250 L), stocked at 25 fish per tank. The tanks were supplied with a mixture of underground and sand-filtered seawater (1:1 ratio) at a flow rate of 4.2 L min^−1^, with continuous aeration. Natural photoperiods (12 h light: 12 h dark) were maintained throughout the experimental period.

Water quality was routinely monitored once a day using a digital metre (AZ-8603; Taichung, Taiwan). During the feeding trial, water temperature, dissolved oxygen, salinity, and pH averaged 20.1 ± 1.49 °C, 7.1 ± 0.55 mg L^−1^, 32.5 ± 0.60 ‰, and 7.1 ± 0.32, respectively (mean ± SD). Uneaten feeds were not collected. The tanks were syphoned daily to remove accumulated wastes and maintain suitable water conditions.

### 2.2. Experimental Diets

Six isoproteic (57.5%) and isolipidic (10.5%) diets were prepared to fulfil the nutritional requirements of olive flounder [47,48] (Table 1). The control (Con) diet contained 45% FM (sardine and anchovy meal = 1:1) with 12% soybean meal, 4.5% wheat gluten, 5.5% soy protein concentrate, 8% tankage meal, and 4.5% poultry by-product meal as the protein sources [49,50]; 3.8% starch and 7% wheat flour for the carbohydrate sources; and 4.3% fish oil as the lipid source. In the Con diet, 10% FM protein (equivalent to 22.2% of FM) was replaced with TBM, BSF, and FSB as a single protein source, and combined TBM and BSF or TBM and FSB at a ratio of 1:1, named the TBM, BSF, FSB, CTB, and CTF diets, respectively. All diets were assigned to triplicate groups of fish.

All feed ingredients were meticulously mixed with water (3:1 weight ratio), extruded into 3 mm pellets using a vertical mixer (B20GA; Eben Commerce, Ansan-si, Gyeonggi-do, Republic of Korea) and a pelleting machine (SMC-32; SL Company, Incheon Metropolitan City, Republic of Korea), dried at 45 °C for 24 h, and kept at −20 °C until feeding. Throughout the 12-week feeding trial, juvenile olive flounder were hand-fed carefully to apparent satiation twice daily (08:00 and 16:00), allowing sufficient time for consumption and minimizing feed waste, ensuring consistent feeding conditions and allowing for accurate comparison of growth performance across dietary treatments.

### 2.3. Evaluation of Performance of Olive Flounder

At the end of the 12-week feeding trial, all surviving fish were fasted for 24 h and then anesthetized using an immersion bath containing tricaine methanesulfonate (50 mg L^−1^). Afterwards, the weight gain (WG) of all live fish in each tank was collectively weighed. The growth performance, biological indices, and feed utilization of fish were calculated according to Sim and Cho’s study [37] as follows: WG (g fish^−1^) = final weight of fish (g) − Initial weight of fish (g), specific growth rate (SGR, % day^−1^) = [Ln final weight of fish (g) − Ln initial weight of fish (g)] × 100/days of the feeding trial (84 days), feed efficiency (FE) = [total final weight of fish (g) − total initial weight of fish (g)]/total FC (g), protein efficiency ratio (PER) = WG of fish (g)/protein consumption of fish (g), protein retention (PR, %) = protein gain of fish (g) × 100/protein consumption of fish (g), condition factor (CF, g cm^−3^) = body weight of fish (g) × 100/total length of fish (cm)^3^, viscerosomatic index (VSI, %) = viscera weight of fish (g) × 100/body weight of fish (g), and hepatosomatic index (HSI, %) = liver weight of fish (g) × 100/body weight of fish (g). The biological indices of fish including CF, VSI, and HSI were determined using ten randomly selected fish from each tank.

### 2.4. Evaluation of Blood Chemistry of Olive Flounder

Blood was collected using a 1 mL syringe pre-coated with heparin from the caudal vein of five randomly selected anesthetized fish from each tank. Plasma samples were prepared by centrifuging whole blood at 2700× *g* at 4 °C for 10 min, after which the supernatant was transferred and kept in a deep freezer at −70 °C for further analysis. Subsequently, plasma biochemical parameters were quantified utilizing an automated dry-chemistry analyzer (FUJI DRI-CHEM NX500i; Fujifilm Corp., Tokyo, Japan). The parameters included aspartate aminotransferase (AST), alanine aminotransferase (ALT), alkaline phosphatase (ALP), total bilirubin (T-BIL), total cholesterol (T-CHO), triglyceride (TG), total protein (TP), and albumin (ALB). Serum samples were collected following the identical protocol used for plasma samples via a syringe (without pre-coated heparin). Following, superoxide dismutase (SOD) was measured using an ELISA Kit (MBS705758; MyBiosource Inc., San Diego, CA, USA) [37] and lysozyme (LYZ) activity was measured using a validated turbidimetric assay [51].

### 2.5. Evaluation of the Biochemical Composition of the Experimental Diets and Fish

The proximate composition of the homogenized experimental diets, initial fish (*n* = 10) prior to the experiment, and remaining fish (≥13 individuals per tank) were analyzed following standard AOAC protocols [52]. For instance, moisture content was measured by drying the samples in a forced-air convection oven (LDO-250F; Dalhan Labtech Co., Ltd., Namyangju-si, Gyeonggi-do, Republic of Korea) at 105 °C for 6 h in the case of the dry samples and 24 h for the wet samples. Crude protein was quantified via the Kjeldahl method using a Kjeltec^TM^ 2100 distillation unit (Foss, Hillerød, Denmark). Crude lipid was extracted utilizing the Soxtec^TM^ 2043 system (Foss, Hillerød, Denmark). Ash content was measured by incinerating the samples in a muffle furnace (MF-14; Han Yang Scientific Equipment Co. Ltd., Seoul Metropolitan City, Republic of Korea) at 550 °C for 4 h. The in vitro pepsin digestibility of the experimental diets was analyzed according to AOAC official method 971.09 [53], by digesting the sample in 0.2% pepsin hydrochloric acid solution, and then filtering the residue through No. 2A filter paper to obtain the crude protein via an auto Kjeldahl apparatus (kjeltec^TM^ 8400, Foss, Hillerød, Denmark) and calculated as: digestibility (%) = [initial protein (%) − residue protein (%)] × 100/initial protein (%).

Amino acids, excluding tryptophan, were quantified by using an automated amino acid analyzer (Hitachi L-8800; Tokyo, Japan). For hydrolysis, 0.2 g of the sample was placed in a sealed tube with 30 mL of 6 M HCl, purged with nitrogen, and heated at 110 °C for 24 h. After hydrolysis, the solution was filtered through a 0.45 µm nylon syringe filter prior to analysis. Tryptophan was assessed distinctly via high-performance liquid chromatography using Sykam S1125 (Sykam GmbH, Germany) under conditions optimized for its preservation and detection.

Fatty acid compositions were determined by comparing sample profiles to a certified FAME standard (FAME mix CRM47885; Supelco^TM^, St. Louis, MO, USA). Total lipids were extracted by applying a 2:1 (*v*/*v*) chloroform–methanol solvent system, in accordance with the classical Folch method widely recognized for its high lipid recovery efficiency [54]. The isolated lipids were then methylated using 14% boron trifluoride in methanol. Gas chromatographic separation and quantification of fatty acid methyl esters were performed using an Agilent HP 6890 system endowed with a capillary column (SP-2560; 100 m × 0.25 mm i.d., 0.20 µm film thickness; Supelco^TM^, St. Louis, MO, USA). Each fatty acid was pinpointed by contrasting its chromatographic retention time against that of known standard compounds.

### 2.6. Economic Evaluation of the Trial

The economic conversion ratio (ECR) and economic profit index (EPI) of the study was conducted according to Martínez-Llorens et al. [55]: ECR (US$ kg^−1^) = FC of fish (kg fish^−1^)/WG (kg fish^−1^) × diet price (US$ kg^−1^) and EPI (US$ fish^−1^) = [final weight (kg fish^−1^) × fish sale price (US$ kg^−1^)] − [ECR (US$ kg^−1^) × WG (kg fish^−1^)]. The unit prices of the feed ingredients used for diet formulation were recorded as follows (US$ kg^−1^; US$ 1 = 1450 KRW): FM 2.21, TBM 1.17, BSF 3.10, FSB 0.97, soybean meal 0.85, starch 3.19, wheat flour 0.46, wheat gluten 1.66, soy protein concentrate 0.83, tankage meal 0.83, poultry by-product meal 0.80, fish oil 2.37, lecithin 4.83, betaine 7.59, taurine 3.59, mono calcium phosphate 4.14, vitamin premix 7.14, mineral premix 5.74, vitamin C 6.07, vitamin E 12.41, and choline 1.24. The diet costs were estimated by applying the market price of each ingredient to its share in the diet formulation. The average selling value of fish was calculated as US$ 13.80 kg^−1^.

### 2.7. Statistical Analysis

IBM SPSS Statistics version 25.0 (IBM Corp., Armonk, NY, USA) was used to perform the statistical analysis. Percentage values were exposed to an arcsine transformation prior to analysis. The assumptions of homogeneity of variances and normality were assessed using Levene’s test and Shapiro–Wilk tests, respectively. The tank was considered as the experimental unit and tank means were used for inferential comparisons where appropriate. Differences among dietary treatments resulting from the 10% substitution of FM protein were evaluated by one-way analysis of variance (ANOVA). If any significant (*p* < 0.05) effect was detected, Tukey’s HSD test was applied for multiple comparisons of means. Principal component analysis (PCA) was additionally executed to summarize multivariate patterns among dietary treatments and to illustrate variation in the parameters (growth, feed utilization, biological indices, blood chemistry, proximate composition, amino acid and fatty acid profiles of fish, and economic analysis). Furthermore, hierarchical clustering analysis (HCA) was applied to evaluate similarity relationships among the diets. All exploratory multivariate analyses, including PCA and HCA, were performed, and corresponding plots, such as PCA biplots and HCA dendrograms, were generated through OriginPro 25 (Northampton, MA, USA). During HCA, variable standardization, distance, and the group average were set as Z scores, Pearson correlation, and clustering, respectively.

## 3. Results

### 3.1. Amino and Fatty Acid Profiles in the Major Protein Ingredients and Feeds

Leucine and lysine were the most abundant IAAs, while aspartic acid and glutamic acid were the most abundant dispensable amino acids (DAAs) in the major protein sources and the experimental diets (Table 2).

Total saturated fatty acid (∑SFA) and total monounsaturated fatty acid (∑MUFA) in FM, TBM, and BSF were relatively higher than those in FSB (Table 3). However, eicosapentaenoic acid (EPA, C20:5n-3), docosahexaenoic acid (DHA, C22:6n-3), and total n-3 highly unsaturated fatty acid (∑n-3 HUFA) in FM and TBM were relatively higher than those in BSF and FSB. Among the main protein ingredients, FM contained the highest ∑n-3 HUFA, which resulted in the highest ∑n-3 HUFA in the Con diet. However, dietary substitution of FM with FSB lowered ∑SFA and ∑MUFA in the FSB and CTF diets, while dietary substitution of FM with TBM, BSF, FSB or their combinations lowered ∑n-3 HUFA.

### 3.2. Performance of Olive Flounder

**Figure 1:**
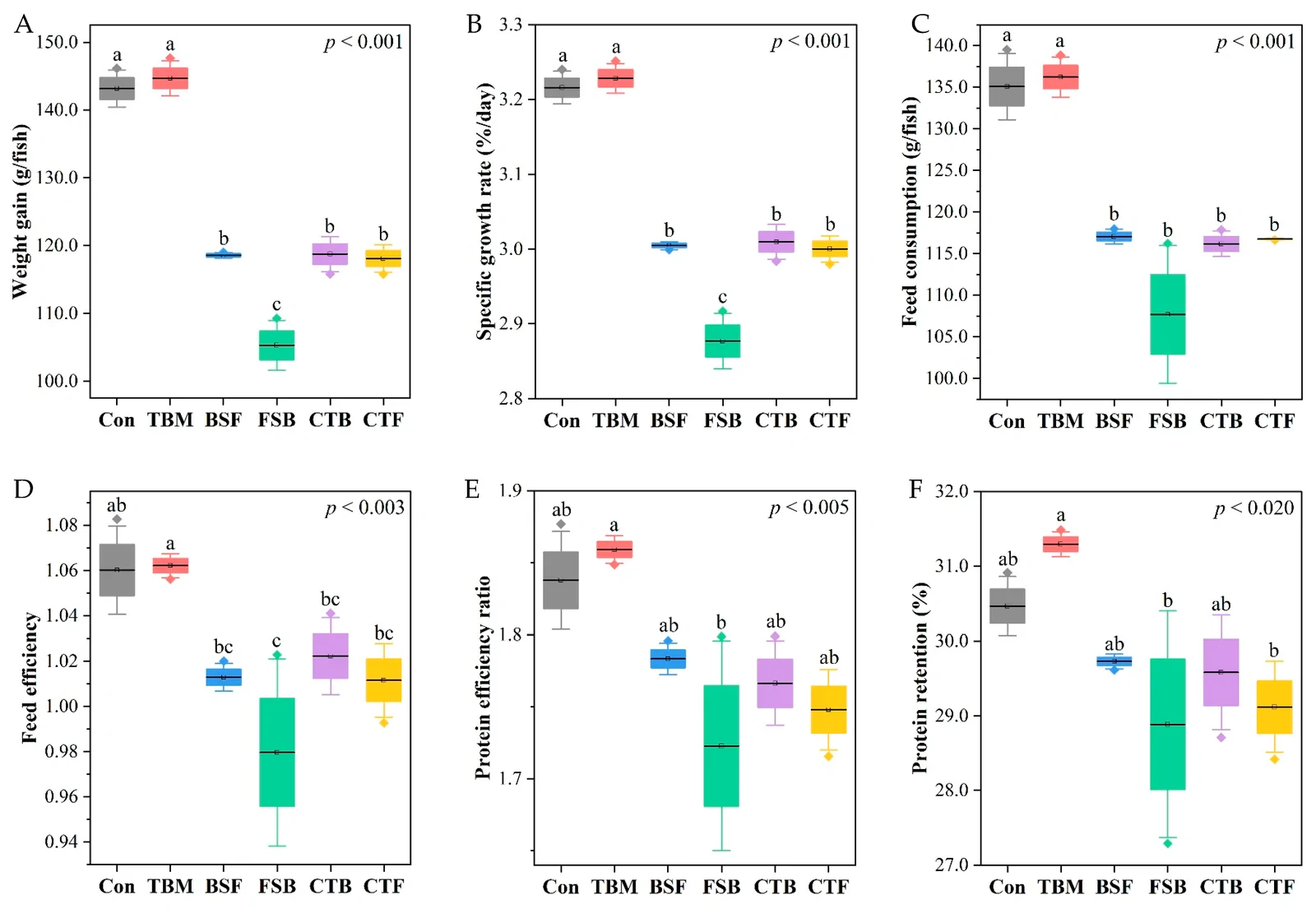
Olive flounder fed the experimental diets [Con, the 45% FM-basal diet; TBM, 10% FM protein replacement with tuna by-product meal; BSF, 10% FM protein replacement with black soldier fly larvae meal; FSB, 10% FM protein replacement with fermented soybean meal; CTB, 10% FM protein replacement with combined tuna by-product meal and black soldier fly larvae meal (1:1); and CTF, 10% FM protein replacement with combined tuna by-product meal and fermented soybean meal (1:1)] for 12 weeks showed box plots of weight gain (g fish^−1^) (**A**), specific growth rate (% day^−1^) (**B**), feed consumption (g fish^−1^) (**C**), feed efficiency (**D**), protein efficiency ratio (**E**), and protein retention (%) (**F**). A different lowercase letter above the box indicates significant differences (*p* < 0.05) according to Tukey’s HSD test (mean of triplicates ± standard error).

The experimental fish all survived at the end of the 12-week feeding trial (Table 4). The WG and SGR were significantly (*p* < 0.001 for both) higher in fish fed the Con (143.2 ± 1.58 g fish^−1^ and 3.22 ± 0.013% day^−1^, mean ± SE) and TBM (144.7 ± 1.50 g fish^−1^ and 3.23 ± 0.011% day^−1^) diets compared to those fed the BSF (118.6 ± 0.24 g fish^−1^ and 3.00 ± 0.003% day^−1^), FSB (105.3 ± 2.13 g fish^−1^ and 2.88 ± 0.021% day^−1^), CTB (118.8 ± 1.49 g fish^−1^ and 3.01 ± 0.013% day^−1^), and CTF (118.1 ± 1.15 g fish^−1^ and 3.00 ± 0.010% day^−1^, respectively) diets (Figure 1A,B). In addition, the WG and SGR of fish fed the BSF, CTB, and CTF diets were also significantly higher than those fed the FSB diet.

The FC was significantly (*p* < 0.001) higher in fish fed the Con (135.1 ± 2.30 g fish^−1^) and TBM (136.2 ± 1.40 g fish^−1^) diets than that of fish fed the BSF (117.1 ± 0.52 g fish^−1^), FSB (107.1 ± 4.77 g fish^−1^), CTB (116.2 ± 0.88 g fish^−1^), and CTF (116.7 ± 0.06 g fish^−1^) diets (Figure 1C). The FE was significantly (*p* < 0.003) higher in fish fed the TBM (1.06 ± 0.003) diet than that of fish fed the BSF (1.01 ± 0.004), FSB (0.98 ± 0.024), CTB (1.02 ± 0.010), and CTF (1.01 ± 0.009) diets, but not significantly (*p* > 0.05) distinct from that of fish fed the Con (1.06 ± 0.011) diet (Figure 1D). Fish fed the TBM diet exhibited a significantly (*p* < 0.005) higher PER (1.86 ± 0.005) than that of fish fed the FSB (1.72 ± 0.042) diet (Figure 1E). Fish fed the TBM diet achieved a significantly (*p* < 0.020) higher PR (31.3 ± 0.10%) than that of fish fed the FSB (28.9 ± 0.88%) and CTF (29.1 ± 0.35%) diets, but not significantly distinct from that of fish fed the Con (30.5 ± 0.23%), BSF (29.7 ± 0.06%), and CTB (29.6 ± 0.44%) diets (Figure 1F).

The CF of fish fed the Con and TBM diets was significantly higher (*p* < 0.001) than that of fish fed the BSF, FSB, CTB, and CTF diets. However, there were no significant differences in both VSI (*p* > 0.620) and HSI (*p* > 0.283) of fish among the experimental diets.

### 3.3. Blood Chemistry of Olive Flounder

Neither the plasma parameters (AST, ALT, ALP, T-BIL, T-CHO, TG, TP, and ALB) nor the serum parameters (SOD and LYZ activity) of the fish were affected by the dietary treatments (Table 5).

### 3.4. Proximate Composition of the Whole-Body Olive Flounder

The whole-body moisture content ranged from 75.8% to 76.2%, crude protein content ranged from 16.5% to 16.7%, crude lipid content ranged from 3.8% to 4.7%, and ash content ranged from 3.5% to 4.1% (Table 6). The proximate composition of the whole-body fish did not significantly (*p* > 0.05) differ among the experimental diets.

### 3.5. Amino Acid and Fatty Acid Profiles of Olive Flounder

No significant (*p* > 0.05) differences in either the IAA or DAA profiles of the whole-body fish were observed among the experimental diets (Table 7). Likewise, the fish fed all experimental diets showed no significant (*p* > 0.05) differences in fatty acid profiles, except for lauric acid (C12:0; *p* < 0.001), stearic acid (C18:0; *p* < 0.021), and erucic acid (C22:1n-9; *p* < 0.027) (Table 8). While C22:1n-9 exhibited a significant (*p* < 0.027) difference in one-way ANOVA, no significant pairwise differences among diets were detected by Tukey’s HSD test.

### 3.6. Economic Evaluation of the Study

The BSF diet exhibited the highest price among the experimental diets (Table 9). The lowest ECR was obtained in the TBM diet, but the highest was for the BSF diet. The Con and TBM diets produced significantly (*p* < 0.001) higher EPI than all other diets.

### 3.7. Multivariate Analysis

**Figure 2:**
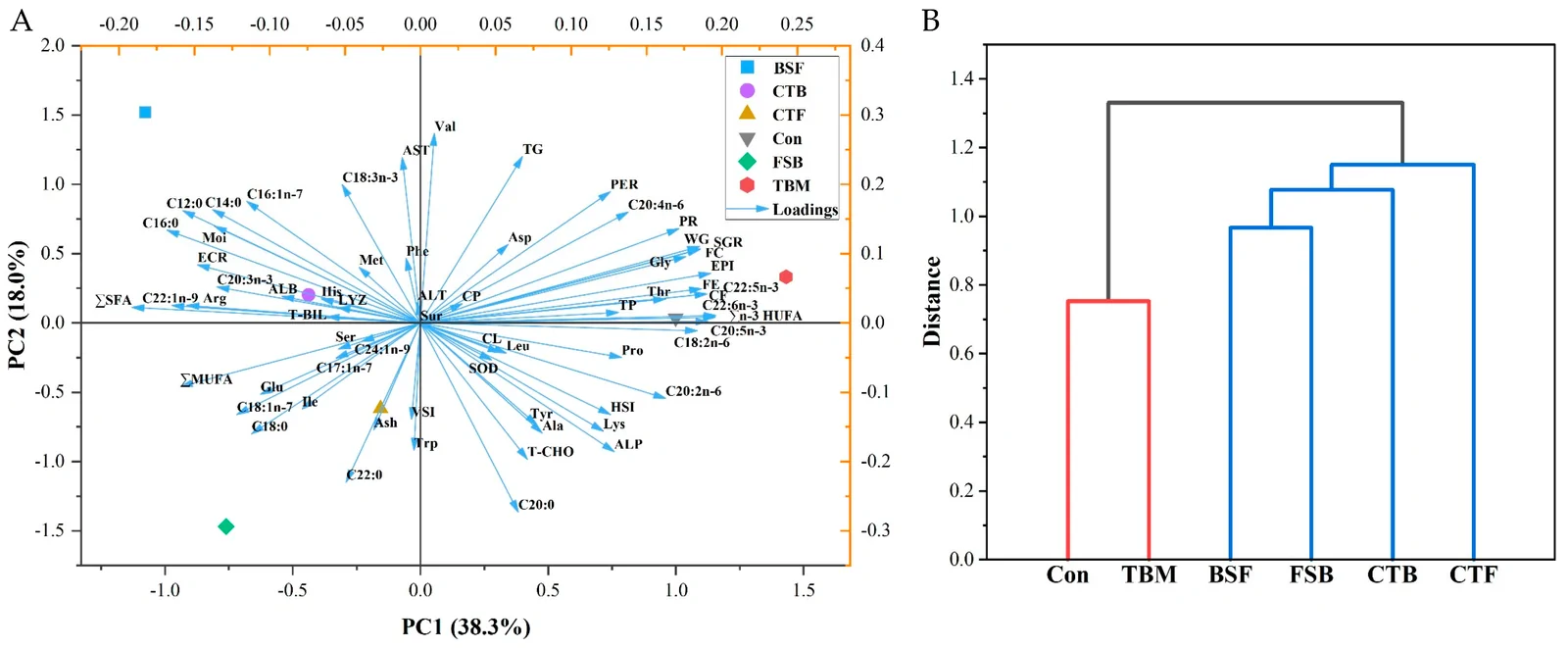
A principal component analysis (PCA) of olive flounder fed the experimental diets [Con, the 45% FM-basal diet; TBM, 10% FM protein replacement with tuna by-product meal; BSF, 10% FM protein replacement with black soldier fly larvae meal; FSB, 10% FM protein replacement with fermented soybean meal; CTB, 10% FM protein replacement with combined tuna by-product meal and black soldier fly larvae meal (1:1); and CTF, 10% FM protein replacement with combined tuna by-product meal and fermented soybean meal (1:1)] for 12 weeks on observed parameters including growth, feed utilization, biological indices, blood chemistry, proximate composition, amino and fatty acid profiles, and economic efficiency; an illustrating PCA biplot of the first two principal components (PC1 38.3% and PC2 18.0%, together 56.3%) (**A**) and similarity relationships among the diets in a hierarchical clustering analysis (HCA) dendrogram (**B**). For HCA, variable standardization, distance, and the group average were set as Z scores, Pearson correlation, and clustering, respectively. Ala, alanine; ALB, albumin; ALP, alkaline phosphatase; ALT, alanine aminotransferase; Arg, arginine; Asp, aspartic acid; AST, aspartate aminotransferase; CF, condition factor; CL, crude lipid; CP, crude protein; FC, feed consumption; FE, feed efficiency; Glu, glutamic acid; Gly, glycine; His, histidine; HSI, hepatosomatic index; Ile, isoleucine; Leu, leucine; Lys, lysine; LYZ, lysozyme activity; Met, methionine; Moi, moisture; PER, protein efficiency ratio, Phe, phenylalanine; PR, protein retention; Pro, proline; Ser, serine; SGR, specific growth rate; SOD, superoxide dismutase; Sur, survival; T-BIL, total bilirubin; T-CHO, total cholesterol; TG, triglyceride; Thr, threonine; TP, total protein; Trp, tryptophan; Tyr, tyrosine; Val, valine; VSI, viscerosomatic index; and WG, weight gain.

The PCA, together with PC1 and PC2, accounted for 56.3% of the total variance, with PC1 contributing 38.3% and PC2 contributing 18.0%, effectively distinguishing the experimental diets (Figure 2A). Distinct clustering was observed among the experimental diets; the BSF, FSB, CTB, and CTF diets grouped on the negative side of PC1, while the TBM and Con diets clustered on the positive side. The WG, SGR, FC, FE, PER, PR, and EPI, as well as fatty acid profiles including linoleic acid (C18:2n-6), eicosadienoic acid (C20:2n-6), arachidonic acid (C20:4n-6), EPA, DHA, and ∑n-3 HUFA, were strongly aligned with TBM and Con diets. In contrast, the BSF, FSB, CTB, and CTF diets were linked with ∑SFA, palmitoleic acid (C16:1n-7), α-linolenic acid (C18:3n-3), eicosatrienoic acid (C20:3n-3), erucic acid (C22:1 n-9), nervonic acid (C24:1n-9), ∑MUFA, and some amino acids, such as arginine, serine, isoleucine, glutamic acid, but showed weaker association with growth parameters.

The hierarchical clustering of the experimental diets revealed two primary groupings (Figure 2B). The Con and TBM diets exhibited close clustering. The second cluster comprised the BSF, FSB, CTB, and CTF diets, with the BSF and FSB diets forming a sub-cluster that subsequently joined with the CTB and CTF diets.

## 4. Discussion

The SGR (3.2% day^−1^) of the fish fed the Con diet in this experiment exceeded the values (3.1% day^−1^ [61], 2.9% day^−1^ [62], and 2.0% day^−1^ [49]) reported in previous studies, in which olive flounder with initial body weights of 14.5, 9.9, and 7.9 g were fed the basal diets containing 60%, 45%, and 70% FM, respectively. These results indicate that the growth performance observed in the present study was satisfactorily achieved. Therefore, evaluating alternative protein sources for juvenile olive flounder using 10% FM protein replacement in a 45% FM-basal diet represents a practical and potential applicable feeding strategy. Several efforts were undertaken to reduce reliance on expensive conventional protein sources, in particular, FM, by substituting partially or entirely with more cost-effective and sustainable alternatives in aquafeeds [17,63,64,65]. Among such alternatives, fishery by-product meal, such as TBM, has gained attention as a potentially sustainable protein source [5,12,66,67]. Similarly, in the present study, fish fed the TBM diet achieved comparable growth performance and feed utilization to those fed the Con diet but exhibited significantly higher growth performance and feed utilization than those fed the BSF and FSB diets or CTB and CTF diets, indicating TBM as a feasible replacer for FM in the olive flounder diet when 10% FM protein is replaced in a 45% FM-basal diet.

The superior growth performance (WG and SGR) of fish fed the Con and TBM diets compared to those fed all other diets was well reflected from the higher FC of fish fed the former compared to those fed the latter in this experiment, agreeing with previous studies, in which higher FC resulted in higher WG and SGR in olive flounder [46], greater amberjack (*Seriola dumerili*) [66], and Asian seabass [68]. This indicates that 10% FM protein can replace TBM in a 45% FM-basal diet, which is equivalent to 22.2% of FM, without impairing the WG, SGR, and FC of olive flounder. Both FM and TBM are enriched with high-quality marine-derived proteins with balanced IAA profiles, together with naturally occurring taurine, nucleotides, and other water-soluble compounds, which can act as feeding stimulants and enhance the palatability of the diet [8,69,70]. This may be one of the contributing factors to why fish fed the Con and TBM diet exhibited higher FC in the current study. Furthermore, the consistent growth performance (WG and SGR) observed in the TBM diet may be attributed to the digestive and metabolic adaptations of olive flounder, which enable the efficient utilization of marine by-products [71,72]. Similar findings with various levels of FM substitution with TBM have been reported in marine finfish diets [14,15,66,73], indicating TBM as a viable replacer for FM. For example, Biswas et al. [66] reported that up to 25% of FM replacement, equivalent to 17.5% FM protein, with TBM in a 70% FM-basal diet could be achieved without a significant reduction in the WG of greater amberjack. Similarly, Kim et al. [41] reported that up to 18% FM protein replacement, equivalent to 30% of FM, with fermented TBM could be achieved in a 60% FM-basal diet without deteriorating the SGR in the 7-week feeding trial. The higher FE of fish provided with a TBM diet compared to that of fish provided with all other diets, except for that of fish provided with the Con diet, might indicate that TBM was the best FM replacer when 10% FM protein was replaced with TBM, BSF, and FSB, and their combinations in a 45% FM-basal diet of olive flounder. Although alternative protein sources were evaluated at the single replacement level (10% FM protein) in a 45% FM-based diet, further studies with multiple replacement levels are required to determine whether the observed advantages of TBM remain consistent across different replacement levels. The lowest FE was obtained in fish fed the FSB diet, attributed to the poorest WG, SGR, and FC in the present experiment.

In this study, nevertheless, all experimental diets met the arginine (2.04–2.10% of the diet) [56] and lysine (1.55–2.16%) [57], threonine (1.03%) [59] and valine (1.77–1.89%) [60] requirements for olive flounder. However, methionine content in all experimental diets, including the Con diet, was relatively lower than the dietary requirement of methionine (1.44–1.49% of the diet in the presence of 0.06% cysteine) for olive flounder [58]. However, the dietary methionine requirement might be spared by cystine [70]. For example, cystine can spare up to 50% of dietary methionine in chinook salmon (*Oncorhynchus tshawytcha*) [70] and in red drum (*Sciaenops ocellatus*) [74]. Since cystine ranged from 0.70% in the BSF diet to 0.77% in the Con diet, possibly the low methionine in all experimental diets might not have negatively affected the growth performance of olive flounder in this study. Additionally, the dietary nutrient requirements of fish are influenced by body size and age, with the methionine requirement generally decreasing as fish grow [75,76]. Alam et al. [58] reported that smaller juvenile (initial weight of 2.8 g) olive flounder required a dietary methionine level of 1.44–1.49%. In this study, however, the larger juvenile (initial weight of 10.3 g) fish likely required a lower dietary methionine level than that previously reported for smaller fish [58].

FM replacements with TBM, BSF, FSB, and their combinations tended to lower ∑n-3 HUFA including EPA and DHA in the TBM, BSF, FSB, CTB, and CTF diets in this experiment. In particular, relatively lower ∑n-3 HUFA in the BSF, FSB, CTB, and CTF diets might have contributed to the deterioration in growth performance of olive flounder. Likewise, lower dietary ∑n-3 HUFA levels led to reduced growth performance in European seabass (*Dicentrarchus labrax*) [77] and large yellow croaker (*Larmichthys crocea*) [78].

Olive flounder fed the TBM diet exhibited the highest PER and PR, whereas those fed the FSB diet demonstrated the lowest values, with all the other diets producing PER and PR comparable to the Con diet. Although fermentation improves nutrient digestibility [27], the lowest PER and PR obtained in fish fed the FSB diet might indicate inefficient utilization of dietary protein, being consistent with previous studies highlighting the dietary limitations of soybean-derived proteins for carnivorous fish [78,79]. Therefore, additional supplementation (e.g., IAAs and/or digestibility-enhancing enzymes or other feed additives) is required when fish are fed with alternative plant protein sources [7]. PR reflects the proportion of ingested protein that is incorporated into body tissues rather than catabolized for energy [80]. Fish primarily excrete nitrogen in the form of ammonia, and when dietary protein quality is insufficient, excessive protein catabolism increases nitrogen excretion, leading to lower PR values [81]. This mechanism may explain why fish fed the FSB and CTF diets produced significantly lower PR in the present study.

The BSF and FSB diets using a single protein replacer led to the inferior growth of olive flounder compared to the Con diet in the present study. A slightly higher replacement of FM protein with BSF and FSB without adversely affecting growth has been observed in various fish species [23,82,83,84]. These differences may be attributed to species-specific nutritional requirements, digestive physiology, and feed palatability, as well as FM inclusion level in the diet [85,86]. Although BSF contains a similar protein level to FM, it often shows deficiencies in amino acid profiles especially for lysine and methionine [87]. Therefore, additional synthetic amino acids are required to overcome these limitations when a diet is formulated with BSF [88]. Even though BSF offers a promising alternative protein source, the presence of chitin may impair digestibility and reduce the efficiency of nutrient absorption when included in aquafeeds [89]. Previously, chitin was identified as one of the major factors of growth inhibition in rainbow trout (*Salmo gairdneri*) [90] and in Atlantic salmon [91]. Alternatively, FSB, while typically superior to unfermented soybean meal, may still carry residual ANFs or lead to imbalanced amino acid digestibility, as observed in plant-derived feed ingredients [92,93]. This may be one of the reasons why BSF and FSB diets showed less favourable growth outcomes in comparison to the TBM diet. This suggests that olive flounder can tolerate moderate FM replacement by marine by-products without adverse effects on growth performance. However, its ability to utilize insect- or plant-derived proteins may be more limited unless they are supplemented with flavour enhancers or processed in the diet [94]. In the present study, the combined diets (CTB and CTF) did not match the efficacy of the TBM diet alone, even though blending is frequently suggested as a strategy to complement nutrient profiles [44,95]. All experimental diets were prepared to meet the known nutrient requirements of olive flounder and consisted of practical ingredients such as poultry by-product meal, tankage, taurine and betaine. Nutrient utilization may have been affected by the variation in protein digestibility, amino acid availability, palatability, and other ingredient-specific characteristics. These results reinforce that ingredient quality and compatibility determine success, even when blending strategies are implemented. Future research should focus on improving the palatability of the experimental diets and include in vivo or in vitro digestibility using multiple enzymes, as well as a quantitative assessment of chitin, ANFs, and relative digestive-physiological parameters to better establish any causal relationship between dietary chitin, ANFs, and the growth performance of fish. In this experiment, fish were hand-fed carefully to apparent satiation to minimize waste. As uneaten feed was not collected, a potential difference among dietary treatments may have influenced FC, feed utilization, and economic analysis in this study. Therefore, future studies should collect and quantify uneaten feed to better assess FC and its implications for feed utilization and economic analysis.

CF serves as a practical indicator for monitoring the nutritional status, physiological condition, and well-being of fish [96]. The VSI is frequently used as an indicator of lipid utilization biomarker in fish and typically increases with increasing dietary lipid levels [97]. The HSI is frequently used to assess fish nutritional condition, acting as an indicator of liver glycogen and glucose reserves [98]. The CF of fish fed the Con and TBM diets remained remarkably higher than that of fish fed the BSF, FSB, CTB, and CTF diets in this experiment, indicating that CF was well reflected in growth performance. However, the VSI and HSI of fish were not affected by dietary treatments [21,29,84]. No significant difference in the VSI and HSI of fish fed the experimental diets may indicate a reflection of isolipidic (10%) contents and similar carbohydrate levels (19.6–22.9%) across the diets, respectively.

Fish blood serves as a reliable indicator for assessing blood cell damage and disorders, reflecting physiological responses to diet, environmental stressors, and overall welfare [99,100]. LYZ activity and SOD are important components of fish innate immunity, where LYZ functions to directly degrade bacterial cell walls while SOD mitigates oxidative stress by converting harmful superoxide radicals into less damaging molecules [101,102]. No significant differences were detected in this study in plasma and serum parameters among the dietary treatments [103,104]. However, reliance solely on plasma or serum biochemistry may overlook subtler effects; thus, further research should include histopathology and possibly metabolomics profiling to identify early or tissue-specific responses.

The biochemical composition, including the proximate composition [34,105], amino acid [12], and fatty acid profiles of the whole-body fish, except for lauric acid, stearic acid, and erucic acid, were not altered by the dietary treatments in this experiment. Although the fish experienced fluctuations in dietary amino acid composition, they normally maintain stable amino acid profiles as long as protein and energy intake remain adequate by adjusting internal amino acid metabolism [106]. In general, the fatty acid profile of fish corresponds to that of their dietary intake, albeit with certain species-and tissue-specific variation [107,108,109]. In this study, relatively higher lauric acid in the BSF diet and stearic acid in the FSB diet led to higher whole-body lauric acid and stearic acid, respectively. Lauric acid is typically absorbed and metabolized differently from long-chain fatty acids [110]. For instance, in rainbow trout fed coconut oil rich in lauric acid, about 57% of the ingested lauric acid was retained, indicating relatively low oxidation rates, which means that its distinctive metabolism allows dietary inclusion to directly elevate whole-body levels [110]. Stearic acid tends to be incorporated more stably into body lipids, as observed in mandarin fish (*Siniperca scherzeri*); diets incorporating lard resulted in significantly higher stearic acid levels in fillet tissues compared to fish oil-based diets [111]. This may suggest that stearic acid levels are highly responsive to dietary consumption and less prone to regulatory homeostasis. Other fatty acids, such as mono- or polyunsaturated fatty acids, are often tightly regulated due to their essential biological roles in membrane fluidity, signalling, and anti-inflammatory fluctuations [112]. Unless diets differ greatly in these fatty acids, their whole-body concentration may remain unchanged, maintained by active metabolic balance and selective tissue retention [113].

Feed cost typically constitutes 40–70% of total aquaculture production expenses, rendering feed management a pivotal determinant of farm profitability [114]. The economic evaluation of the feeding trial was a scenario-based ingredient-cost comparison in the current study. The ingredient prices used in this study may vary with origin, sources, and country-to-country specific specifications, quality, or quantity, and the fish selling price may vary with size, seasons, demand, and locations; hence, they should be considered with caution for further interpretation. A relatively higher BSF price led to a higher BSF diet cost in the present study. The lowest ECR was obtained by the TBM diet, indicating greater economic efficiency than the other diets. The EPI offers a useful metric to evaluate the cost-effectiveness of feeds, as it integrates important variables such as the WG, FC, feed cost, and sell value of fish. In the current study, fish fed the Con and TBM diets resulted in significantly higher EPI than the other diets, indicating TBM as a viable alternative protein source for olive flounder when 10% FM protein is replaced in a 45% FM-basal diet. Similarly, Baek and Cho [14] found that replacing 40% of FM with TBM in a 55% FM-basal diet of red sea bream maintained growth performance, FC, and feed utilization, and resulted in the highest EPI for farmers. Therefore, from an economic viewpoint, the use of TBM as a partial replacement for FM constitutes a promising strategy to reduce feed cost and enhance the profit margin of aquaculture operations without compromising biological performance.

According to the exploratory multivariate analysis, the TBM diet was the most effective among the experimental diets, demonstrating a strong positive correlation with growth performance (WG and SGR), feed utilization, and beneficial fatty acids, including linoleic acid, eicosadienoic acid, arachidonic acid, EPA, DHA, and ∑n-3 HUFA as well as EPI. These findings indicate that TBM promotes both growth and feed utilization, making it the superior diet for olive flounder at 10% FM protein replacement in a 45% FM-basal diet.

## 5. Conclusions

Among the five tested formulations, TBM can replace 10% FM protein in a 45% FM-basal diet without negatively affecting the growth performance, feed utilization, blood chemistry, and biochemical composition of olive flounder, resulting in a higher EPI for farmers. Therefore, TBM is a promising partial FM substitute (at 10% FM protein replacement) in the tested 45% FM-based diet of olive flounder. Further long-term feeding trials with multiple replacement levels along with molecular approaches should be considered for TBM as a partial FM replacer for sustainable olive flounder aquaculture.

## Figures and Tables

**Table 1:** Ingredients and chemical composition of experimental diets (%, dry matter basis).

	Experimental Diets					
	Con	TBM	BSF	FSB	CTB	CTF
Ingredients (%)						
Fish meal ^1^	45.0	35.0	35.0	35.0	35.0	35.0
Tuna by-product meal ^2^		11.6			5.8	5.8
Black soldier fly larvae meal ^3^			13.6		6.8	
Fermented soybean meal ^4^				11.1		5.5
Soybean meal	12.0	12.0	12.0	12.0	12.0	12.0
Starch	3.8	2.3	1.1	1.9	1.7	2.1
Wheat flour	7.0	7.0	7.0	7.0	7.0	7.0
Wheat gluten	4.5	4.5	4.5	4.5	4.5	4.5
Soy protein concentrate	5.5	5.5	5.5	5.5	5.5	5.5
Tankage meal	8.0	8.0	8.0	8.0	8.0	8.0
Poultry by-product meal	4.5	4.5	4.5	4.5	4.5	4.5
Fish oil	4.3	4.2	3.4	5.1	3.8	4.7
Lecithin	0.5	0.5	0.5	0.5	0.5	0.5
Betaine	1.0	1.0	1.0	1.0	1.0	1.0
Taurine	0.5	0.5	0.5	0.5	0.5	0.5
Monocalcium phosphate	0.7	0.7	0.7	0.7	0.7	0.7
Vitamin premix ^5^	1.0	1.0	1.0	1.0	1.0	1.0
Mineral premix ^6^	1.0	1.0	1.0	1.0	1.0	1.0
Vitamin C	0.1	0.1	0.1	0.1	0.1	0.1
Vitamin E	0.1	0.1	0.1	0.1	0.1	0.1
Choline	0.5	0.5	0.5	0.5	0.5	0.5
Nutrients (%)						
Dry matter	96.5	95.8	95.8	96.0	96.3	96.0
Crude protein	57.7	57.1	56.9	56.8	57.9	57.9
Crude lipid	10.8	10.8	10.5	10.6	10.9	10.7
Ash	11.2	10.9	12.1	9.7	11.6	10.6
Carbohydrate ^7^	20.3	21.2	20.5	22.9	19.6	20.3
0.2% pepsin digestibility (%)	93.3	93.3	90.6	92.9	92.6	93.3

^1^ Fish meal [FM (sardine and anchovy meal = 1:1): crude protein (CP) 73.3%, crude lipid (CL) 8.6%, and ash 15.0%] was imported from Chile. ^2^ Tuna by-product meal (TBM: CP 63.1%, CL 8.5%, and ash 20.3%) was purchased from Woojin Feed Ind. Co. Ltd., Incheon Metropolitan City, Korea. ^3^ Black soldier fly larvae meal (BSF: CP 53.9%, CL 12.8%, and ash 24.9%) was supplied by CIEF Co. Ltd., Gimge, Jeonbuk, Korea. ^4^ Fermented soybean meal (FSB: CP 65.7%, CL 0.5%, and ash 5.9%) was bought from CJ CheilJedang, Seoul Metropolitan City, Korea. ^5^ Vitamin premix (g kg^−1^ mix): DL-α-tocophyryl acetate, 18.8; L-ascorbic acid, 121.2; thiamine hydrochloride, 2.7; pyridoxine hydrochloride, 1.8; riboflavin, 9.1; niacin, 36.4; myo-inositol, 181.8; Ca-D-pantothenate, 12.7; D-biotin, 0.27; p-aminobenzoic acid, 18.2; folic acid, 0.68; menadione, 1.8; cholecalciferol, 0.003; retinyl acetate, 0.73; and cyanocobalamin, 0.003. ^6^ Mineral premix (g kg^−1^ mix): NaH_2_PO_4_2H_2_O, 370.0; MgSO_4_7H_2_O, 80.0; ZnSO_4_7H_2_O, 20.0; ferric citrate, 40.0; CuCl, 0.2; Ca-lactate, 356.5; AlCl_3_6H_2_O, 0.15; KI, 0.15; MnSO_4_H_2_O, 2.0; Na_2_Se_2_O_3_, 0.01; and CoCl_2_6H_2_O, 1.0. ^7^ Carbohydrate was calculated by the difference [Carbohydrate = 100 − (CP + CL + ash)].

**Table 2:** The amino acid profiles (% of the diet) of the main protein ingredients and the experimental diets.

Parameters	Main Protein Ingredients				Requirement	Experimental Diets					
	FM	TBM	BSF	FSB		Con	TBM	BSF	FSB	CTB	CTF
Indispensable amino acids (IAAs)											
Arginine	4.08	3.63	2.28	4.52	2.04–2.10 ^1^	3.32	3.25	3.13	3.37	3.15	3.34
Histidine	1.69	2.01	1.36	1.56		1.27	1.27	1.19	1.24	1.22	1.25
Isoleucine	2.69	2.28	1.79	2.56		2.08	2.00	1.94	2.05	1.95	2.01
Leucine	5.01	4.15	2.92	4.00		3.92	3.78	3.65	3.86	3.71	3.80
Lysine	5.56	5.16	3.02	4.61	1.55–2.16 ^2^	3.80	3.61	3.46	3.54	3.54	3.57
Methionine	1.13	1.47	0.71	1.10	1.44–1.49 ^3^	1.20	1.22	1.03	1.14	1.09	1.16
Phenylalanine	2.78	2.39	1.85	2.87		2.26	2.21	2.13	2.30	2.15	2.23
Threonine	3.10	2.73	1.95	2.44	1.03 ^4^	2.25	2.17	2.09	2.15	2.10	2.15
Tryptophan	1.14	1.00	1.10	1.21		0.52	0.52	0.49	0.54	0.51	0.52
Valine	3.37	2.91	2.58	2.89	1.77–1.89 ^5^	2.54	2.43	2.36	2.43	2.41	2.42
∑IAA ^6^	30.55	27.73	19.56	27.76		23.16	22.46	21.47	22.62	21.83	22.45
Dispensable amino acids (DAAs)											
Alanine	4.37	4.17	3.08	3.25		3.23	3.11	2.95	2.96	3.01	3.05
Aspartic acid	6.36	5.42	4.26	5.58		4.87	4.69	4.53	4.87	4.60	4.77
Cystine	1.24	0.67	0.58	1.30		0.77	0.76	0.70	0.76	0.73	0.75
Glutamic acid	9.05	7.55	6.07	9.15		8.37	8.24	7.92	8.69	8.06	8.33
Glycine	4.26	4.59	2.52	3.84		3.62	3.66	3.32	3.40	3.51	3.47
Proline	3.14	3.32	3.18	3.89		2.92	2.98	2.92	3.01	2.97	2.98
Serine	2.95	2.61	2.19	3.02		2.34	2.31	2.25	2.40	2.26	2.32
Tyrosine	1.84	1.37	2.45	2.85		1.35	1.30	1.61	1.39	1.49	1.56
∑DAA ^7^	33.21	29.70	24.33	32.88		27.47	27.05	26.20	27.48	26.63	27.23

In the main protein ingredients: FM, fish meal; TBM, tuna by-product meal; BSF, black soldier fly larvae meal; and FSB, fermented soybean meal. In the experimental diets: Con, the 45% FM-basal diet; TBM, 10% FM protein replacement with tuna by-product meal; BSF, 10% FM protein replacement with black soldier fly larvae meal; FSB, 10% FM protein replacement with fermented soybean meal; CTB, 10% FM protein replacement with combined tuna by-product meal and black soldier fly larvae meal (1:1); and CTF, 10% FM protein replacement with combined tuna by-product meal and fermented soybean meal (1:1). ^1^ Arginie [56]. ^2^ Lysine [57]. ^3^ Methionine [58]. ^4^ Threonine [59]. ^5^ Valine [60]. ^6^ ∑IAA: total indispensable amino acids. ^7^ ∑DAA: total dispensable amino acids.

**Table 3:** The fatty acid profiles (% of total fatty acids) of the main protein ingredients and the experimental diets.

Parameters	Main Protein Ingredients				Experimental Diets					
	FM	TBM	BSF	FSB	Con	TBM	BSF	FSB	CTB	CTF
C12:0	0.07	0.06	22.20	0.25	0.26	0.20	3.84	0.23	2.20	0.17
C14:0	6.18	4.74	4.44	0.30	3.21	2.90	3.59	2.68	3.05	2.69
C16:0	22.34	24.05	16.75	17.23	22.00	22.35	17.66	18.02	19.43	19.24
C18:0	3.72	4.30	4.31	4.80	5.23	5.51	5.55	5.69	5.44	5.32
C20:0	2.51	1.74	0.34	0.34	1.68	2.23	0.84	1.61	1.73	2.18
C22:0	0.22	0.44	0.06	0.46	0.18	0.13	0.20	0.21	0.14	0.19
∑SFA ^1^	35.04	35.33	48.10	23.38	32.56	33.32	31.68	28.44	31.99	29.79
C16:1n-7	6.53	5.21	2.97	0.33	5.91	4.78	4.25	2.57	4.52	4.10
C17:1n-7	1.08	1.13	0.24	0.06	0.54	0.71	0.19	0.09	0.45	0.41
C18:1n-7	15.47	20.37	23.82	17.01	23.45	26.02	25.3	21.3	25.78	23.13
C22:1n-9	0.07	0.11	0.15	0.00	0.26	0.28	0.32	0.20	0.30	0.20
C24:1n-9	0.63	1.05	0.00	0.00	0.02	0.04	0.05	0.06	0.04	0.05
∑MUFA ^2^	23.78	27.87	27.18	17.40	30.18	31.83	30.11	24.22	31.09	27.89
C18:2n-6	4.54	3.15	17.84	30.56	12.9	13.45	26.39	30.63	18.13	23.28
C18:3n-3	2.31	2.92	0.52	6.75	4.44	4.79	3.20	4.18	4.08	4.09
C20:2n-6	0.25	0.45	0.07	0.00	0.28	0.51	0.16	0.15	0.42	0.44
C20:3n-3	1.62	4.22	0.37	0.15	0.11	0.12	0.13	0.12	0.13	0.13
C20:4n-6	0.50	0.53	0.03	0.10	0.35	0.32	0.28	0.30	0.30	0.35
C20:5n-3	13.40	4.39	1.18	0.35	7.81	4.50	2.18	2.14	4.21	4.05
C22:5n-3	2.63	1.02	0.00	0.64	1.48	1.34	1.11	1.21	1.25	1.28
C22:6n-3	12.15	13.21	0.02	0.08	9.29	9.23	4.07	8.05	7.82	8.07
∑n-3 HUFA ^3^	29.80	22.84	1.57	1.22	18.69	15.19	7.49	11.52	13.41	13.53
Unknown	3.78	6.91	4.69	20.54	0.58	0.59	0.66	0.55	0.58	0.63

In the main protein ingredients: FM, fish meal; TBM, tuna by-product meal; BSF, black soldier fly larvae meal; and FSB, fermented soybean meal. In the experimental diets: Con, the 45% FM-basal diet; TBM, 10% FM protein replacement with tuna by-product meal; BSF, 10% FM protein replacement with black soldier fly larvae meal; FSB, 10% FM protein replacement with fermented soybean meal; CTB, 10% FM protein replacement with combined tuna by-product meal and black soldier fly larvae meal (1:1); and CTF, 10% FM protein replacement with combined tuna by-product meal and fermented soybean meal (1:1). ^1^ ∑SFA: total saturated fatty acids. ^2^ ∑MUFA: total monounsaturated fatty acids. ^3^ ∑n-3 HUFA: total n-3 highly unsaturated fatty acids.

**Table 4:** The survival (%), condition factor (CF, g cm^−3^), viscerosomatic index (VSI, %), and hepatosomatic index (HSI, %) of olive flounder fed the experimental diets for 12 weeks.

Parameters	Experimental Diets						*p*-Value
	Con	TBM	BSF	FSB	CTB	CTF	
IW (g fish^−1^)	10.3 ± 0.01	10.3 ± 0.00	10.3 ± 0.01	10.3 ± 0.00	10.3 ± 0.02	10.3 ± 0.01	
FW (g fish^−1^)	153.5 ± 1.58 ^a^	155.0 ± 1.50 ^a^	128.9 ± 0.24 ^b^	115.6 ± 2.14 ^c^	129.1 ± 1.50 ^b^	128.4 ± 1.16 ^b^	*p* < 0.001
Survival (%)	100.0 ± 00	100.0 ± 00	100.0 ± 00	100.0 ± 00	100.0 ± 00	100.0 ± 00	
CF (g cm^−3^)	1.1 ± 0.00 ^a^	1.1 ± 0.01 ^a^	1.0 ± 0.01 ^b^	1.0 ± 0.01 ^b^	1.0 ± 0.00 ^b^	1.0 ± 0.01 ^b^	*p* < 0.001
VSI (%)	4.1 ± 0.13	4.6 ± 0.42	4.3 ± 0.04	4.6 ± 0.27	4.3 ± 0.11	4.5 ± 0.17	*p* > 0.620
HSI (%)	1.3 ± 0.01	1.4 ± 0.13	1.2 ± 0.02	1.3 ± 0.07	1.2 ± 0.03	1.4 ± 0.10	*p* > 0.283

Con, the 45% FM-basal diet; TBM, 10% FM protein replacement with tuna by-product meal; BSF, 10% FM protein replacement with black soldier fly larvae meal; FSB, 10% FM protein replacement with fermented soybean meal; CTB, 10% FM protein replacement with combined tuna by-product meal and black soldier fly larvae meal (1:1); and CTF, 10% FM protein replacement with combined tuna by-product meal and fermented soybean meal (1:1). IW, initial weight; FW, final weight; CF, condition factor; VSI, viscerosomatic index; and HSI, hepatosomatic index. Values (mean of triplicates ± standard error) with different lowercase superscripts in the same row indicate significant differences (*p* < 0.05) according to one-way ANOVA followed by Tukey’s HSD test.

**Table 5:** Blood biochemistry of olive flounder fed experimental diets for 12 weeks.

Parameters	Experimental Diets						*p*-Value
	Con	TBM	BSF	FSB	CTB	CTF	
Plasma parameters							
AST (U L^−1^)	14.4 ± 0.06	14.9 ± 0.22	15.0 ± 0.17	14.4 ± 0.06	14.8 ± 0.06	14.3 ± 0.19	*p* > 0.451
ALT (U L^−1^)	6.2 ± 0.15	6.6 ± 0.06	6.6 ± 0.11	6.6 ± 0.06	5.9 ± 0.06	6.1 ± 0.15	*p* > 0.209
ALP (U L^−1^)	117.0 ± 0.58	117.9 ± 0.97	115.3 ± 0.92	117.2 ± 0.34	115.9 ± 0.64	117.8 ± 0.06	*p* > 0.692
T-BIL (U L^−1^)	0.7 ± 0.03	0.6 ± 0.01	0.7 ± 0.01	0.7 ± 0.01	0.6 ± 0.01	0.6 ± 0.02	*p* > 0.966
T-CHO (mg dL^−1^)	355.8 ± 5.80	354.1 ± 3.79	342.2 ± 1.60	358.9 ± 6.39	356.1 ± 3.12	347.9 ± 3.11	*p* > 0.752
TG (mg dL^−1^)	454.2 ± 2.47	469.8 ± 5.64	468.0 ± 7.20	443.6 ± 1.56	449.4 ± 2.36	445.3 ± 9.43	*p* > 0.442
TP (g dL^−1^)	5.9 ± 0.12	5.6 ± 0.06	5.5 ± 0.07	5.4 ± 0.06	5.4 ± 0.04	5.7 ± 0.08	*p* > 0.218
ALB (g dL^−1^)	1.1 ± 0.04	1.0 ± 0.01	1.1 ± 0.04	1.1 ± 0.03	1.2 ± 0.03	1.0 ± 0.03	*p* > 0.616
Serum parameters							
SOD (ng mL^−1^)	73.0 ± 0.74	71.2 ± 2.36	71.2 ± 0.86	72.9 ± 0.54	65.4 ± 2.74	69.5 ± 2.07	*p* > 0.503
LYZ (U mL^−1^)	4.6 ± 0.25	5.3 ± 0.18	5.0 ± 0.30	4.9 ± 0.33	5.5 ± 0.52	5.2 ± 0.14	*p* > 0.601

Con, the 45% FM-basal diet; TBM, 10% FM protein replacement with tuna by-product meal; BSF, 10% FM protein replacement with black soldier fly larvae meal; FSB, 10% FM protein replacement with fermented soybean meal; CTB, 10% FM protein replacement with combined tuna by-product meal and black soldier fly larvae meal (1:1); and CTF, 10% FM protein replacement with combined tuna by-product meal and fermented soybean meal (1:1). AST, aspartate aminotransferase; ALT, alanine aminotransferase; ALP, alkaline phosphatase; T-BIL, total bilirubin; T-CHO, total cholesterol; TG, triglyceride; TP, total protein; ALB, albumin; SOD, superoxide dismutase; and LYZ, lysozyme activity. The values (mean of triplicates ± standard error) were presented.

**Table 6:** The proximate composition (%, wet weight) of the whole-body olive flounder fed the experimental diets for 12 weeks.

Parameters	Experimental Diets						*p*-Value
	Con	TBM	BSF	FSB	CTB	CTF	
Moisture	75.8 ± 0.02	75.9 ± 0.13	76.2 ± 0.20	75.9 ± 0.08	76.0 ± 0.15	76.1 ± 0.09	*p* > 0.316
Crude protein	16.5 ± 0.05	16.7 ± 0.02	16.6 ± 0.05	16.6 ± 0.12	16.6 ± 0.14	16.6 ± 0.04	*p* > 0.401
Crude lipid	4.5 ± 0.35	4.6 ± 0.22	4.5 ± 0.21	4.5 ± 0.36	3.8 ± 0.24	4.7 ± 0.22	*p* > 0.327
Ash	3.6 ± 0.24	3.8 ± 0.30	3.6 ± 0.23	4.1 ± 0.36	3.8 ± 0.30	3.5 ± 0.07	*p* > 0.538

Con, the 45% FM-basal diet; TBM, 10% FM protein replacement with tuna by-product meal; BSF, 10% FM protein replacement with black soldier fly larvae meal; FSB, 10% FM protein replacement with fermented soybean meal; CTB, 10% FM protein replacement with combined tuna by-product meal and black soldier fly larvae meal (1:1); and CTF, 10% FM protein replacement with combined tuna by-product meal and fermented soybean meal (1:1). The values (mean of triplicates ± standard error) were presented.

**Table 7:** The amino acid profiles (% of wet weight) of the whole-body olive flounder fed the experimental diets for 12 weeks.

Parameters	Experimental Diets						*p*-Value
	Con	TBM	BSF	FSB	CTB	CTF	
Indispensable amino acids (IAAs)							
Arginine	9.9 ± 0.25	9.9 ± 0.21	10.1 ± 0.27	10.0 ± 0.30	10.2 ± 0.22	10.2 ± 0.15	*p* > 0.916
Histidine	5.4 ± 0.07	5.2 ± 0.12	5.3 ± 0.15	5.3 ± 0.11	5.4 ± 0.11	5.2 ± 0.14	*p* > 0.853
Isoleucine	6.2 ± 0.03	6.2 ± 0.13	6.3 ± 0.08	6.3 ± 0.18	6.2 ± 0.17	6.3 ± 0.06	*p* > 0.984
Leucine	12.6 ± 0.28	12.7 ± 0.17	12.5 ± 0.19	12.7 ± 0.21	12.5 ± 0.34	12.5 ± 0.39	*p* > 0.995
Lysine	17.5 ± 0.37	17.4 ± 0.23	17.3 ± 0.08	17.4 ± 0.26	17.4 ± 0.15	17.4 ± 0.12	*p* > 0.997
Methionine	7.6 ± 0.02	7.6 ± 0.05	7.7 ± 0.10	7.7 ± 0.03	7.5 ± 0.18	7.6 ± 0.12	*p* > 0.973
Phenylalanine	8.4 ± 0.15	8.4 ± 0.26	8.4 ± 0.29	8.4 ± 0.11	8.4 ± 0.09	8.4 ± 0.12	*p* > 0.999
Threonine	21.6 ± 0.52	21.6 ± 0.05	21.5 ± 0.25	21.5 ± 0.04	21.6 ± 0.36	21.6 ± 0.54	*p* > 0.999
Tryptophan	0.9 ± 0.03	0.9 ± 0.01	0.9 ± 0.07	1.0 ± 0.02	0.9 ± 0.01	0.9 ± 0.02	*p* > 0.904
Valine	9.8 ± 0.22	9.9 ± 0.14	9.9 ± 0.12	9.7 ± 0.06	9.9 ± 0.18	9.8 ± 0.23	*p* > 0.975
Dispensable amino acids (DAAs)							
Alanine	36.9 ± 0.73	36.7 ± 0.36	36.6 ± 0.45	36.6 ± 0.17	36.8 ± 0.24	36.7 ± 0.60	*p* > 0.994
Aspartic acid	3.2 ± 0.05	3.3 ± 0.15	3.3 ± 0.13	3.2 ± 0.03	3.3 ± 0.07	3.2 ± 0.06	*p* > 0.981
Glutamic acid	20.5 ± 0.44	20.9 ± 0.11	20.9 ± 0.17	20.9 ± 0.11	20.9 ± 0.29	21.0 ± 0.41	*p* > 0.907
Glycine	12.3 ± 0.22	12.3 ± 0.27	12.2 ± 0.25	12.1 ± 0.18	12.2 ± 0.09	12.2 ± 0.19	*p* > 0.982
Proline	4.7 ± 0.11	4.7 ± 0.16	4.7 ± 0.11	4.7 ± 0.14	4.7 ± 0.11	4.7 ± 0.08	*p* > 0.999
Serine	18.2 ± 0.51	18.0 ± 0.58	18.2 ± 0.20	18.2 ± 0.15	17.9 ± 0.21	17.9 ± 0.44	*p* > 0.956
Tyrosine	4.2 ± 0.12	4.2 ± 0.12	4.2 ± 0.05	4.2 ± 0.10	4.2 ± 0.08	4.2 ± 0.03	*p* > 0.999

Con, the 45% FM-basal diet; TBM, 10% FM protein replacement with tuna by-product meal; BSF, 10% FM protein replacement with black soldier fly larvae meal; FSB, 10% FM protein replacement with fermented soybean meal; CTB, 10% FM protein replacement with combined tuna by-product meal and black soldier fly larvae meal (1:1); and CTF, 10% FM protein replacement with combined tuna by-product meal and fermented soybean meal (1:1). The values (mean of triplicates ± standard error) were presented.

**Table 8:** The fatty acid profiles (% of total fatty acids) of the olive flounder fed the experimental diets for 12 weeks.

Parameters	Experimental Diets						*p*-Value
	Con	TBM	BSF	FSB	CTB	CTF	
C12:0	0.0 ± 0.00 ^c^	0.0 ± 0.00 ^c^	2.0 ± 0.11 ^a^	0.7 ± 0.37 ^bc^	1.1 ± 0.03 ^b^	0.0 ± 0.05 ^c^	*p* < 0.001
C14:0	3.2 ± 0.12	3.1 ± 0.23	4.0 ± 0.52	3.2 ± 0.19	3.8 ± 0.45	3.7 ± 0.41	*p* > 0.396
C16:0	17.0 ± 0.39	16.5 ± 1.27	21.0 ± 1.23	18.1 ± 1.48	18.0 ± 1.59	18.6 ± 1.89	*p* > 0.336
C18:0	5.7 ± 0.27 ^bc^	5.3 ± 0.26 ^c^	6.2 ± 0.50 ^bc^	7.8 ± 0.67 ^a^	5.4 ± 0.34 ^c^	5.7 ± 0.57 ^bc^	*p* < 0.021
C20:0	2.7 ± 0.42	2.7 ± 0.28	1.7 ± 0.25	3.2 ± 0.79	2.2 ± 0.38	2.9 ± 0.89	*p* > 0.496
C22:0	0.2 ± 0.17	0.1 ± 0.13	0.1 ± 0.07	0.4 ± 0.17	0.3 ± 0.17	0.4 ± 0.06	*p* > 0.469
∑SFA ^1^	28.8 ± 0.62	27.7 ± 1.82	35.0 ± 2.25	33.4 ± 1.44	30.8 ± 2.04	31.3 ±2.33	*p* > 0.126
C16:1n-7	4.5 ± 0.19	4.3 ± 0.21	5.0 ± 0.19	4.3 ± 0.20	4.7 ± 0.28	4.8 ± 0.43	*p* > 0.350
C17:1n-7	0.1 ± 0.06	0.1 ± 0.06	0.1 ± 0.04	0.2 ± 0.09	0.2 ± 0.01	0.0 ± 0.00	*p* > 0.175
C18:1n-7	25.0 ± 0.59	25.9 ± 1.61	26.2 ± 0.66	27.9 ± 0.37	28.6 ± 1.07	27.8 ± 1.60	*p* > 0.217
C22:1n-9	0.1 ± 0.06	0.1 ± 0.11	0.6 ± 0.10	0.4 ± 0.10	0.2 ± 0.16	0.5 ± 0.09	*p* < 0.027
C24:1n-9	0.5 ± 0.10	0.6 ± 0.10	0.6 ± 0.14	0.7 ± 0.34	0.5 ± 0.08	0.4 ± 0.07	*p* > 0.836
∑MUFA ^2^	30.1 ± 0.69	30.9 ± 1.68	32.5 ± 0.57	33.6 ± 0.59	34.1 ± 1.34	33.5 ± 2.01	*p* > 0.237
C18:2n-6	23.1 ± 0.46	22.3 ± 1.13	19.4 ± 1.41	19.8 ± 2.64	19.0 ± 0.38	20.6 ± 2.31	*p* > 0.446
C18:3n-3	4.7 ± 0.57	5.0 ± 0.43	5.4 ± 0.29	4.8 ± 0.69	5.2 ± 0.61	4.2 ± 0.11	*p* > 0.585
C20:2n-6	0.8 ± 0.12	0.8 ± 0.10	0.2 ± 0.07	0.5 ± 0.15	0.7 ± 0.32	0.7 ± 0.11	*p* > 0.172
C20:3n-3	0.2 ± 0.11	0.1 ± 0.13	0.4 ± 0.02	0.3 ± 0.19	0.1 ± 0.10	0.2 ± 0.09	*p* > 0.523
C20:4n-6	1.1 ± 0.13	1.2 ± 0.05	1.1 ± 0.11	1.0 ± 0.12	1.0 ± 0.12	1.0 ± 0.13	*p* > 0.713
C20:5n-3	2.8 ± 0.63	2.9 ± 0.82	0.4 ± 0.09	0.7 ± 0.36	1.9 ± 0.62	1.6 ± 0.84	*p* > 0.077
C22:5n-3	1.3 ± 0.13	1.4 ± 0.03	1.2 ± 0.11	1.2 ± 0.03	1.2 ± 0.02	1.2 ± 0.04	*p* > 0.405
C22:6n-3	7.0 ± 0.81	7.7 ± 1.29	4.3 ± 0.03	4.6 ± 0.18	5.9 ± 0.75	5.7 ± 1.06	*p* > 0.085
∑n-3 HUFA ^3^	11.2 ± 1.41	12.1 ± 2.02	6.3 ± 0.17	6.9 ± 0.24	9.2 ± 1.45	8.7 ± 1.91	*p* > 0.075

Con, the 45% FM-basal diet; TBM, 10% FM protein replacement with tuna by-product meal; BSF, 10% FM protein replacement with black soldier fly larvae meal; FSB, 10% FM protein replacement with fermented soybean meal; CTB, 10% FM protein replacement with combined tuna by-product meal and black soldier fly larvae meal (1:1); and CTF, 10% FM protein replacement with combined tuna by-product meal and fermented soybean meal (1:1). The values (mean of triplicates ± standard error) with different lowercase superscripts in the same row indicate significant difference (*p* < 0.05) according to one-way ANOVA followed by Tukey’s HSD test. Although there is a significant (*p* < 0.027) difference in C22:1n-9 in one-way ANOVA, no significant pairwise differences among diets were detected by Tukey’s HSD test. ^1^ ∑SFA: total saturated fatty acids. ^2^ ∑MUFA: total monounsaturated fatty acids. ^3^ ∑n-3 HUFA: total n-3 highly unsaturated fatty acids.

**Table 9:** An economic evaluation of the feeding trial for 12 weeks.

Parameters	Experimental Diets						*p*-Value
	Con	TBM	BSF	FSB	CTB	CTF	
Diet price (US$ kg^−1^)	1.9	1.7	2.0	1.7	1.9	1.7	
ECR ^1^ (US$ kg^−1^)	1.8 ± 0.02 ^b^	1.6 ± 0.00 ^c^	1.9 ± 0.01 ^a^	1.8 ± 0.04 ^b^	1.8 ± 0.02 ^b^	1.7 ± 0.02 ^bc^	*p* < 0.001
EPI ^2^ (US$ fish^−1^)	1.9 ± 0.02 ^a^	1.9 ± 0.02 ^a^	1.5 ± 0.00 ^b^	1.4 ± 0.02 ^c^	1.6 ± 0.02 ^b^	1.6 ± 0.02 ^b^	*p* < 0.001

Con, the 45% FM-basal diet; TBM, 10% FM protein replacement with tuna by-product meal; BSF, 10% FM protein replacement with black soldier fly larvae meal; FSB, 10% FM protein replacement with fermented soybean meal; CTB, 10% FM protein replacement with combined tuna by-product meal and black soldier fly larvae meal (1:1); and CTF, 10% FM protein replacement with combined tuna by-product meal and fermented soybean meal (1:1). The average selling price of olive flounder was calculated as US$ 13.80 kg^−1^ (US$ 1 = 1450 KRW). The values (mean of triplicates ± standard error) with different lowercase superscripts in the same row indicate significant differences (*p* < 0.05) according to one-way ANOVA followed by Tukey’s HSD test. ^1^ Economic conversion ratio (ECR, US$ kg^−1^) = feed consumption of fish (kg fish^−1^)/weight gain (kg fish^−1^) × diet price (US$ kg^−1^). ^2^ Economic profit index (EPI, US$ fish^−1^) = [final weight (kg fish^−1^) × fish sale price (US$ kg^−1^)] − [ECR (US$ kg^−1^) × weight gain (kg fish^−1^)].

## Data Availability

The raw data supporting the conclusions of this article will be made available by the authors on request.

## References

[1] Froese R., Pauly D. (2025). FishBase. World Register of Marines Species (WORMS), World Wide Web Electronic Publication. https://www.fishbase.org.

[2] Hamidoghli A., Won S., Lee S., Lee S., Farris N.W., Bai S.C. (2020). Nutrition and feeding of olive flounder *Paralichthys olivaceus*: A review. Rev. Fish. Sci. Aquac..

[3] KOSIS (2025). Korean Statistical Information Service, Korea. https://kosis.kr/statHtml/statHtml.do?orgId=101&tblId=DT_1EZ0007&conn_path=I2.

[4] Dar S.A., Kole S., Shin S., Jeong H., Jung S. (2021). Comparative study on antigen persistence and immunoprotective efficacy of intramuscular and intraperitoneal injections of squalene—Aluminium hydroxide (Sq + Al) adjuvanted viral hemorrhagic septicaemia virus vaccine in olive flounder (*Paralichthys olivaceus*). Vaccine.

[5] Stieglitz J.D., Hoenig R.H., Baggett J.K., Tudela C.E., Mathur S.K., Benetti D.D. (2021). Advancing production of marine fish in the United States: Olive flounder, *Paralichthys olivaceus*, aquaculture. J. World Aquac. Soc..

[6] Tacon A.G.J., Metian M. (2008). Global overview on the use of fish meal and fish oil in industrially compounded aquafeeds: Trends and future prospects. Aquaculture.

[7] Daniel N. (2018). A review on replacing fish meal in aqua feeds using plant protein sources. Int. J. Fish. Aquat. Stud..

[8] Tacon A.G.J., Metian M. (2015). Feed matters: Satisfying the feed demand of aquaculture. Rev. Fish. Sci. Aquac..

[9] FAO (2024). The State of World Fisheries and Aquaculture 2024—Blue Transformation in Action.

[10] Naghdi S., Rezaei M., Heidari M.G., Tahergorabi R., Lorenzo J.M., Mirzaei F. (2024). Insights into fishery by-product application in aquatic feed and food: A review. Aquac. Int..

[11] Guerard F., Guimas L., Binet A. (2002). Production of tuna waste hydrolysates by a commercial neutral protease preparation. J. Mol. Catal. B Enzym..

[12] Kim K., Jang J.W., Kim K., Lee B., Hur S.W., Han H. (2018). Tuna by-product meal as a dietary protein source replacing fishmeal in juvenile Korean rockfish *Sebastes schlegeli*. Fish. Aquat. Sci..

[13] Li R., Cho S.H. (2023). Substitution impact of tuna by-product meal for fish meal in the diets of rockfish (*Sebastes schlegeli*) on growth and feed availability. Animals.

[14] Baek S., Cho S.H. (2024). Dietary replacement effect of fish meal by tuna by-product meal on growth and feed availability of red sea bream (*Pagrus major*). Animals.

[15] Hernández C., Hardy R.W., Contreras-Rojas D., López-Molina B., González-Rodríguez B., Domínguez-Jimenez P. (2014). Evaluation of tuna by-product meal as a protein source in feeds for juvenile spotted rose snapper *Lutjanus guttatus*. Aquac. Nutr..

[16] Bruno D., Cuff J., Adamaki-Sotiraki C., Ameixa O., Athanassiou C., Boukrouh S., Castellanos F., Gebiola M., Guillaume J., Ibikunle O. (2026). Knowledge gaps in feeding physiology, microbiome and behaviour of insects for food and feed: Overcoming barriers to advancing insect-rearing through interdisciplinarity, standardisation, and emerging technologies. J. Insects Food Feed.

[17] Nogales-Mérida S., Gobbi P., Józefiak D., Mazurkiewicz J., Dudek K., Rawski M., Kierończyk B., Józefiak A. (2019). Insect meals in fish nutrition. Rev. Aquac..

[18] Mohan K., Karthick Rajan D., Muralisankar T., Ganesan A.R., Sathishkumar P., Revathi N. (2022). Use of black soldier fly (*Hermetia illucens* L.) larvae meal in aquafeeds for a sustainable aquaculture industry: A review of past and future needs. Aquaculture.

[19] Eide L.H., Rocha S.D.C., Morales-Lange B., Kuiper R.V., Dale O.B., Djordjevic B., Hooft J.M., Øverland M. (2024). Black soldier fly larvae (*Hermetia illucens*) meal is a viable protein source for Atlantic salmon (*Salmo salar*) during a large-scale controlled field trial under commercial-like conditions. Aquaculture.

[20] Le Boucher R., Chung W., Ng Kai Lin J., Tan L.S.E., Lee C.S. (2024). Black soldier fly larvae meal vs. soy protein concentrate meal: A comparative digestibility study in barramundi (*Lates calcarifer*). Aquac. Nutr..

[21] Wang G., Peng K., Hu J., Yi C., Chen X., Wu H., Huang Y. (2019). Evaluation of defatted black soldier fly (*Hermetia illucens* L.) larvae meal as an alternative protein ingredient for juvenile Japanese seabass (*Lateolabrax japonicus*) diets. Aquaculture.

[22] Zhao J., Pan J., Zhang Z., Chen Z., Mai K., Zhang Y. (2023). Fishmeal protein replacement by defatted and full-fat black soldier fly larvae meal in juvenile turbot diet: Effects on the growth performance and intestinal microbiota. Aquac. Nutr..

[23] Li X., Qin C., Fang Z., Sun X., Shi H., Wang Q., Zhao H. (2022). Replacing dietary fish meal with defatted black soldier fly (*Hermetia illucens*) larvae meal affected growth, digestive physiology and muscle quality of tongue sole (*Cynoglossus semilaevis*). Front. Physiol..

[24] Mugwanya M., Dawood M.A.O., Kimera F., Sewilam H. (2023). Replacement of fish meal with fermented plant proteins in the aquafeed industry: A systematic review and meta-analysis. Rev. Aquac..

[25] Siddik M.A.B., Julien B.B., Islam S.M.M., Francis D.S. (2024). Fermentation in aquafeed processing: Achieving sustainability in feeds for global aquaculture production. Rev. Aquac..

[26] de Oliveira N.S., Ha N., da Cunha L., Cipriani L.A., Neto A.T., Skoronski E., Gisbert E., Perez Fabregat T.E.H. (2022). Fermentation of soybean meal with *Lactobacillus acidophilus* allows greater inclusion of vegetable protein in the diet and can reduce *Vibrionacea* in the intestine of the South American catfish (*Rhamdia quelen*). Animals.

[27] Yao Y., Li H., Li J., Zhu B., Gao T. (2021). Anaerobic solid-state fermentation of soybean meal with *Bacillus sp.* to improve nutritional quality. Front. Nutr..

[28] Zheng L., Li D., Li Z., Kang L., Jiang Y., Liu X., Chi Y., Li Y., Wang J. (2017). Effects of *Bacillus* fermentation on the protein microstructure and anti-nutritional factors of soybean meal. Lett. Appl. Microbiol..

[29] Zhou F., Song W., Shao Q., Peng X., Xiao J., Hua Y., Owari B.N., Zhang T., Ng W. (2011). Partial Replacement of fish meal by fermented soybean meal in diets for black sea bream, *Acanthopagrus schlegelii*, juveniles. J. World Aquac. Soc..

[30] Zhang Q., Guo M., Li F., Qin M., Yang Q., Yu H., Xu J., Liu Y., Tong T. (2023). Evaluation of fermented soybean meal to replace a portion fish meal on growth performance, antioxidant capacity, immunity, and mTOR signaling pathway of Coho salmon (*Oncorhynchus kisutch*). Aquac. Nutr..

[31] Weng L., Wang Z., Zhuang W., Yang T., Xu X., Liu J., Xu Z., Chen R., Wang Q., Wang S. (2023). Effect of replacing fish meal using fermented soybean meal on growth performance, intestine bacterial diversity, and key gene expression of largemouth bass (*Micropterus salmoides*). Fermentation.

[32] Yang H., Bian Y., Huang L., Lan Q., Ma L., Li X., Leng X. (2022). Effects of replacing fish meal with fermented soybean meal on the growth performance, intestinal microbiota, morphology and disease resistance of largemouth bass (*Micropterus salmoides*). Aquac. Rep..

[33] Lin H., Chen X., Chen S., Zhuojia L., Huang Z., Niu J., Wu K., Lu X. (2012). Replacement of fish meal with fermented soybean meal in practical diets for pompano *Trachinotus ovatus*. Aquac. Res..

[34] Oncul F.O., Aya F.A., Hamidoghli A., Won S., Lee G., Han K.R., Bai S.C. (2019). Effects of the dietary fermented tuna by-product meal on growth, blood parameters, nonspecific immune response, and disease resistance in juvenile olive flounder, *Paralichthys olivaceus*. J. World Aquac. Soc..

[35] Uyan O., Koshio S., Teshima S., Ishikawa M., Thu M., Alam M.S., Michael F.R. (2006). Growth and phosphorus loading by partially replacing fishmeal with tuna muscle by-product powder in the diet of juvenile Japanese flounder, *Paralichthys olivaceus*. Aquaculture.

[36] Lin H., Deng Y., Zhu D., Yang Q., Zhou X., Tan B., Feng L., Chi S. (2023). Effects of partially replacing fishmeal with corn gluten meal on growth, feed utilization, digestive enzyme activity, and apparent nutrient digestibility for juvenile white shrimp, *Litopenaeus vannamei*. Front. Vet. Sci..

[37] Sim Y.J., Cho S.H. (2025). Effect of partial or complete substitution of fish meal by meat meal in the feed of red sea bream (*Pagrus major*) on the growth performance and feed utilization. Aquac. Nutr..

[38] Cai W., Liu H., Han D., Zhu X., Jin J., Yang Y., Xie S. (2022). Complete replacement of fishmeal with plant protein ingredients in gibel carp (*Carassius auratus gibelio*) diets by supplementation with essential amino acids without negative impact on growth performance and muscle growth-related biomarkers. Front. Mar. Sci..

[39] Moutinho S., Martínez-Llorens S., Tomás-Vidal A., Jover-Cerdá M., Oliva-Teles A., Peres H. (2017). Meat and bone meal as partial replacement for fish meal in diets for gilthead seabream (*Sparus aurata*) juveniles: Growth, feed efficiency, amino acid utilization, and economic efficiency. Aquaculture.

[40] Macusi E.D., Cayacay M.A., Borazon E.Q., Sales A.C., Habib A., Fadli N., Santos M.D. (2023). Protein fishmeal replacement in aquaculture: A systematic review and implications on growth and adoption viability. Sustainability.

[41] Kim H.S., Jung W., Myung S.H., Cho S.H., Kim D.S. (2014). Substitution effects of fishmeal with tuna byproduct meal in the diet on growth, body composition, plasma chemistry and amino acid profiles of juvenile olive flounder (*Paralichthys olivaceus*). Aquaculture.

[42] Azarm H.M., Lee S. (2014). Evaluation of fermented soybean meal to replace fish meal for juvenile olive flounder, *Paralichthys olivaceus*: Growth performance, amino acid profile and biochemical parameters. Euro. J. Zool. Res..

[43] Song S., Lim H., Lee K. (2023). Evaluation of black soldier fly *Hermetia illucens* and mealworm *Tenebrio molitor* as a fish meal substitute in a low-fish meal diet for juvenile olive flounder *Paralichthys olivaceus*. Korean J. Fish. Aquat. Sci..

[44] Kader M.A., Koshio S. (2012). Effect of composite mixture of seafood by-products and soybean proteins in replacement of fishmeal on the performance of red sea bream, *Pagrus major*. Aquaculture.

[45] Rimoldi S., Di Rosa A.R., Armone R., Chiofalo B., Hasan I., Saroglia M., Kalemi V., Terova G. (2024). The replacement of fish meal with poultry by-product meal and insect exuviae: Effects on growth performance, gut health and microbiota of the European seabass, *Dicentrarchus labrax*. Microorganisms.

[46] Sim Y.J., Cho S.H., Kim T. (2024). Fish meal substitution effects with the combined animal proteins in the feeds of olive flounder (*Paralichthys olivaceus*) on growth performance, feed availability, and disease resistance against *Streptococcus iniae*. Animals.

[47] Kim K.W., Wang X.J., Bai S.C. (2002). Optimum dietary protein level for maximum growth of juvenile olive flounder *Paralichthys olivaceus* (Temminck et Schlegel). Aquac. Res..

[48] Lee S., Cho S.H., Kim K. (2000). Effects of dietary protein and energy levels on growth and body composition of juvenile flounder *Paralichthys olivaceus*. J. World Aquac. Soc..

[49] Jo S., Park S., Lee S., Tran B.T., Kim J.S., Song J., Lee B., Hur S., Nam T., Lee K. (2021). Effect of low-fishmeal diets on some digestive physiological responses of juvenile and growing olive flounder (*Paralichthys olivaceus*) fed at an industrial-scale fish farm. Aquac. Rep..

[50] Lim H., Kim S., Hur S., Lee S., Kim K., Lee K. (2024). Evaluation of low-fish meal diets containing animals byproducts and plant protein sources for juvenile olive flounder *Paralichthys olivaceus*. Aquac. Int..

[51] Ellis A.E., Stolen J.S., Fletcher T.C., Anderson D.P., Roberson B.S., van Muiswinkel W.B. (1990). Lysozyme assays. Techniques in Fish Immunology.

[52] AOAC (1990). Official Methods of Analysis.

[53] Latimer G.W., AOAC International (2023). AOAC official method 971.09 pepsin digestibility of animal protein feeds: Filtration method. Official Methods of Analysis of AOAC International.

[54] Folch J., Lees M., Stanley G.H.S. (1957). A simple method for the isolation and purification of total lipides from animal tissues. J. Biol. Chem..

[55] Martínez-Llorens S., Moñino A.V., Tomás Vidal A., Salvador V.J.M., Pla Torres M., Jover Cerdá M. (2007). Soybean meal as a protein source in gilthead sea bream (*Sparus aurata* L.) diets: Effects on growth and nutrient utilization. Aquac. Res..

[56] Alam M.S., Teshima S., Koshio S., Ishikawa M. (2002). Arginine requirement of juvenile Japanese flounder *Paralichthys olivaceus* estimated by growth and biochemical parameters. Aquaculture.

[57] Forster I., Ogata H.Y. (1998). Lysine requirement of juvenile Japanese flounder *Paralichthys olivaceus* and juvenile red sea bream *Pagrus major*. Aquaculture.

[58] Alam M.S., Teshima S., Ishikawa M., Koshio S. (2000). Methionine requirement of juvenile Japanese flounder *Paralichthys olivaceus*. J. World Aquac. Soc..

[59] Hasanthi M., Kim M., Lim H., Lim J., Hur S., Lee S., Lee B., Kim K., Lee K. (2023). The dietary requirement for threonine in juvenile olive flounder (*Paralichthys olivaceus*). Fish. Aquat. Sci..

[60] Shin J., Ko D., Hasanthi M., Eom G., Lee K. (2024). Dietary valine requirement of juvenile olive flounder (*Paralichthys olivaceus*). Aquac. Nutr..

[61] Lee J., Choi I.C., Kim K.T., Cho S.H., Yoo J.Y. (2012). Response of dietary substitution of fishmeal with various protein sources on growth, body composition and blood chemistry of olive flounder (*Paralichthys olivaceus*, Temminck & Schlegel, 1846). Fish Physiol. Biochem..

[62] Back S., Park S., Moon J., Lee S., Jo S., Nam T., Bai S.C., Kong I., Lee K., Lee B. (2020). The effects of dietary heat-killed probiotics bacteria additives in low-fishmeal feed on growth performance, immune responses, and intestinal morphology in juvenile olive flounder *Paralichthys olivaceus*. Aquac. Rep..

[63] Gamboa-Delgado J., Márquez-Reyes J.M. (2018). Potential of microbial-derived nutrients for aquaculture development. Rev. Aquac..

[64] Qian Y., Limbu S.M., Qiao F., Luo Y., Chen L., Zhang M., Du Z. (2024). Seeking the best alternatives: A systematic review and meta-analysis on replacing fishmeal with plant protein sources in carnivorous fish species. Rev. Aquac..

[65] Serra V., Pastorelli G., Tedesco D.E.A., Turin L., Guerrini A. (2024). Alternative protein sources in aquafeed: Current scenario and future perspectives. Vet. Anim. Sci..

[66] Biswas A., Shirakawa S., Okimura S., Honryo T., Tanaka H. (2024). Effects of tuna by-product meal on growth, whole-body mercury, phosphorus load, and plasma chemistry in juvenile greater amberjack *Seriola dumerili*. Animals.

[67] Jimenez-Champi D., Romero-Orejon F.L., Muñoz A.M., Ramos-Escudero F. (2024). The revalorization of fishery by-products: Types, bioactive compounds, and food applications. Int. J. Food Sci..

[68] Tola S., Sommit N., Seel-audom M., Khamtavee P., Waiho K., Boonmee T., Yuangsoi B., Munpholsri N. (2022). Effects of dietary tuna hydrolysate supplementation on feed intake, growth performance, feed utilization and health status of Asian sea bass (*Lates calcarifer*) fed a low fish meal soybean meal-based diet. Aquac. Res..

[69] Chotikachinda R., Tantikitti C., Benjakul S., Rustad T., Kumarnsit E. (2013). Production of protein hydrolysates from skipjack tuna (*Katsuwonus pelamis*) viscera as feeding attractants for Asian seabass (*Lates calcarifer*). Aquac. Nutr..

[70] NRC (National Research Council) (1993). Nutrient Requirements of Fish.

[71] Khosravi S., Bui H.T.D., Rahimnejad S., Herault M., Fournier V., Kim S., Jeong J., Lee K. (2015). Dietary supplementation of marine protein hydrolysates in fish-meal based diets for red sea bream (*Pagrus major*) and olive flounder (*Paralichthys olivaceus*). Aquaculture.

[72] Rahman M.M., Kim K.W., Lee S.M. (2022). Apparent digestibility coefficients of animal feed ingredients for olive flounder (*Paralichthys olivaceus*). Fish. Aquat. Sci..

[73] Jeon G.H., Kim H.S., Myung S.H., Cho S.H. (2014). The effect of the dietary substitution of fishmeal with tuna by-product meal on growth, body composition, plasma chemistry and amino acid profiles of juvenile Korean rockfish (*Sebastes schlegeli*). Aquac. Nutr..

[74] Goff J.B., Gatlin D.M. (2004). Evaluation of different sulfur amino acid compounds in the diet of red drum, *Sciaenops ocellatus*, and sparing value of cystine for methionine. Aquaculture.

[75] Einen O., Roem A.J. (1997). Dietary protein/energy ratios for Atlantic salmon in relation to fish size: Growth, feed utilization and slaughter quality. Aquac. Nutr..

[76] Kaushik S.J., Cravedi J.P., Lalles J.P., Sumpter J., Fauconneau B., Laroche M. (1995). Partial or total replacement of fish meal by soybean protein on growth, protein utilization, potential estrogenic or antigenic effects, cholesterolemia and flesh quality in rainbow trout, *Oncorhynchus mykiss*. Aquaculture.

[77] Skalli A., Robin J.H. (2004). Requirement of n-3 long chain polyunsaturated fatty acids for European sea bass (*Dicentrarchus labrax*) juveniles: Growth and fatty acid composition. Aquaculture.

[78] Zuo R., Ai Q., Mai K., Xu W., Wang J., Xu H., Liufu Z., Zhang Y. (2012). Effects of dietary n-3 highly unsaturated fatty acids on growth, nonspecific immunity, expression of some immune related genes and disease resistance of large yellow croaker (*Larmichthys crocea*) following natural infestation of parasites (*Cryptocaryon irritans*). Fish Shellfish Immunol..

[79] Refstie S., Korsøen Ø.J., Storebakken T., Baeverfjord G., Lein I., Roem A.J. (2000). Differing nutritional responses to dietary soybean meal in rainbow trout (*Oncorhynchus mykiss*) and Atlantic salmon (*Salmo salar*). Aquaculture.

[80] Kaushik S.J. (1996). Protein nutrition and metabolism in fish. Protein Metabolism and Nutrition.

[81] Ip A.Y., Chew S.F. (2010). Ammonia production, excretion, toxicity, and defense in fish: A review. Front. Physiol..

[82] Lee S., Azarm H.M., Chang K.H. (2016). Effects of dietary inclusion of fermented soybean meal on growth, body composition, antioxidant enzyme activity and disease resistance of rockfish (*Sebastes schlegeli*). Aquaculture.

[83] Magalhães R., Sánchez-López A., Leal R.S., Martínez-Llorens S., Oliva-Teles A., Peres H. (2017). Black soldier fly (*Hermetia illucens*) pre-pupae meal as a fish meal replacement in diets for European seabass (*Dicentrarchus labrax*). Aquaculture.

[84] Xiao X., Jin P., Zheng L., Zheng L., Cai M., Yu Z., Yu J., Zhang J. (2018). Effects of black soldier fly (*Hermetia illucens*) larvae meal protein as a fishmeal replacement on the growth and immune index of yellow catfish (*Pelteobagrus fulvidraco*). Aquac. Res..

[85] Gougbedji A., Detilleux J., Lalèyè P.A., Francis F., Caparros Megido R. (2022). Can insect meal replace fishmeal? A meta-analysis of the effects of black soldier fly on fish growth performances and nutritional values. Animals.

[86] Siddiqui S.A., Gadge A.S., Hasan M., Rahayu T., Povetkin S.N., Fernando I., Castro-Muñoz R. (2024). Future opportunities for products derived from black soldier fly (BSF) treatment as animal feed and fertilizer—A systematic review. Environ. Dev. Sustain..

[87] Dîrvariu L., Barbacariu C., Burducea M., Simeanu D. (2025). Feed sources for sustainable aquaculture: Black soldier fly larvae (BSFL). Fishes.

[88] Andleeb S., Ahmad I., Asimi O.A., Bazaz A.I., Shah A., Qazi D., Razak I.N., Baba S.H. (2025). Contemporary overview of insect meal in aquaculture: Opportunity & prospects. Proc. Zool. Soc..

[89] Kroeckel S., Harjes A.E., Roth I., Katz H., Wuertz S., Susenbeth A., Schulz C. (2012). When a turbot catches a fly: Evaluation of a pre-pupae meal of the black soldier fly (*Hermetia illucens*) as fish meal substitute—Growth performance and chitin degradation in juvenile turbot (*Psetta maxima*). Aquaculture.

[90] Lindsay G.J.H. (1984). Adsorption of rainbow trout (*Salmo gairdneri*) gastric and chitinase lysozymes by cellulose and chitin. Aquaculture.

[91] Olsen R.E., Suontama J., Langmyhr E., Mundheim H., Ringo E., Melle W., Malde M.K., Hemre G.I. (2006). The replacement of fish meal with Antarctic krill, *Euphausia superba* in diets for Atlantic salmon, *Salmo salar*. Aquac. Nutr..

[92] Francis G., Makkar H.P.S., Becker K. (2001). Antinutritional factors present in plant-derived alternate fish feed ingredients and their effects in fish. Aquaculture.

[93] Gatlin D.M., Barrows F.T., Brown P., Dabrowski K., Gaylord T.G., Hardy R.W., Herman E., Hu G., Krogdahl Å., Nelson R. (2007). Expanding the utilization of sustainable plant products in aquafeeds: A review. Aquac. Res..

[94] Hardy R.W. (2010). Utilization of plant proteins in fish diets: Effects of global demand and supplies of fishmeal. Aquac. Res..

[95] Kader M.A., Koshio S., Ishikawa M., Yokoyama S., Bulbul M., Nguyen B.T., Gao J., Laining A. (2012). Can fermented soybean meal and squid by-product blend be used as fishmeal replacements for Japanese flounder (*Paralichthys olivaceus*)?. Aquac. Res..

[96] Bagenal T.B., Tesch F.W. (1978). Age and growth. Methods for Assessment of Fish Production in Fresh Waters.

[97] Wang J.T., Han T., Li X.Y., Yang Y.X., Yang M., Hu S.X., Jiang Y.D., Harpaz S. (2017). Effects of dietary protein and lipid levels with different protein-to-energy ratios on growth performance, feed utilization and body composition of juvenile red-spotted grouper, *Pinephelus akaara*. Aquac. Nutr..

[98] Chellappa S., Huntingford F.A., Strang R.H.C., Thomson R.Y. (1995). Condition factor and hepatosomatic index as estimates of energy status in male three-spined stickleback. J. Fish Biol..

[99] Ahmed I., Reshi Q.M., Fazio F. (2020). The influence of the endogenous and exogenous factors on hematological parameters in different fish species: A review. Aquac. Int..

[100] Shahjahan M., Islam M.J., Hossain M.T., Mishu M.A., Hasan J., Brown C. (2022). Blood biomarkers as diagnostic tools: An overview of climate-driven stress responses in fish. Sci. Total Environ..

[101] Martínez-Álvarez R.M., Morales A.E., Sanz A. (2005). Antioxidant defenses in fish: Biotic and abiotic factors. Rev. Fish Biol. Fish..

[102] Saurabh S., Sahoo P.K. (2008). Lysozyme: An important defence molecule of fish innate immune system. Aquac. Res..

[103] Kim M., Lim H., Lee B., Hur S., Lee S., Kim K., Lee K. (2020). Replacing fish meal with a mixture of plant and animal protein sources in the diets of juvenile olive flounder *Paralichthys olivaceus*. Korean J. Fish. Aquat. Sci..

[104] Li R., Cho S.H., Kim T. (2023). Effect of replacing dietary fish meal protein with combined animal meals on the growth performance of olive flounder (*Paralichthys olivaceus*). Aquac. Rep..

[105] Takakuwa F., Tanabe R., Nomura S., Inui T., Yamada S., Biswas A., Tanaka H. (2022). Availability of black soldier fly meal as an alternative protein source to fish meal in red sea bream (*Pagrus major*, Temminck & Schlegel) fingerling diets. Aquac. Res..

[106] Wilson R.P., Halver J.E. (1986). Protein and amino acid requirements of fishes. Annu. Rev. Nutr..

[107] Böhm M., Schultz S., Koussoroplis A., Kainz M.J. (2014). Tissue-specific fatty acids response to different diets in common carp (*Cyprinus carpio* L.). PLoS ONE.

[108] Caballero M.J., Obach A., Rosenlund G., Montero D., Gisvold M., Izquierdo M.S. (2002). Impact of different dietary lipid sources on growth, lipid digestibility, tissue fatty acid composition and histology of rainbow trout, *Oncorhynchus mykiss*. Aquaculture.

[109] Xu H., Turchini G.M., Francis D.S., Liang M., Mock T.S., Rombenso A., Ai Q. (2020). Are fish what they eat? A fatty acid’s perspective. Prog. Lipid Res..

[110] Figueiredo-Silva A.C., Kaushik S., Terrier F., Schrama J.W., Médale F., Geurden I. (2012). Link between lipid metabolism and voluntary food intake in rainbow trout fed coconut oil rich in medium-chain TAG. Br. J. Nutr..

[111] Sankian Z., Khosravi S., Kim Y., Lee S. (2019). Total replacement of dietary fish oil with alternative lipid sources in a practical diet for mandarin fish, *Siniperca scherzeri*, juveniles. Fish. Aquat. Sci..

[112] Abbott S.K., Else P.L., Atkins T.A., Hulbert A.J. (2012). Fatty acid composition of membrane bilayers: Importance of diet polyunsaturated fat balance. Biochim. Biophys. Acta-Biomembr..

[113] Chen C., Tian H., Liu X., Dai Y., Wen X., Zhao H., Wu K. (2024). Effects of dietary lipid levels on growth, lipid metabolism, fatty acid composition and antioxidant capacity of juvenile greasyback shrimp (*Metapenaeus ensis*). Aquac. Rep..

[114] O’Donncha F., Grant J. (2019). Precision aquaculture. IEEE Internet Things Mag..

